# Isotopic Fractionation and Masking Effects during Biotransformation of Chlorinated Ethanes

**DOI:** 10.1021/acs.est.5c14089

**Published:** 2026-05-28

**Authors:** Elizabeth Phillips, Joan De Vera, Steffen Kümmel, Olivia Bulka, Weibin Chen, Elizabeth Edwards, Ivonne Nijenhuis, Matthias Gehre, Barbara Sherwood Lollar

**Affiliations:** † Department of Earth Sciences, 7938 University of Toronto, 22 Ursula Franklin Street, Toronto, Ontario M5S 3B1, Canada; ∥ The Grantham Institute for Climate Change and the Environment, Imperial College London, Exhibition Road, London SW7 2AZ, U.K.; § Department of Chemical Engineering and Applied Chemistry, University of Toronto, 200 College Street, Toronto, Ontario M5S 3E5, Canada; ‡ Department of Technical Biogeochemistry, Helmholtz Centre for Environmental Research—UFZ, Permoserstrasse 15, Leipzig 04318, Germany; # Institut de Physique du Globe de Paris (IPGP), Université Paris Cité, 1 Rue Jussieu, Paris Cedex 05, 75238, France

**Keywords:** CSIA, contaminant hydrogeology, biotransformation, reductive dehalogenation, enzyme kinetics

## Abstract

Chlorinated ethanes, such as 1,1,1-trichloroethane (1,1,1-TCA) and 1,1-dichloroethane (1,1-DCA), are common groundwater contaminants that undergo abiotic and biotic transformation, although the specific mechanisms remain unresolved. Compound-specific isotope analysis (CSIA) can provide insight into transformation mechanisms, but its application to chlorinated ethanes has been limited compared to the extensive literature on chlorinated ethenes. This study presents the first dual-element stable isotope (carbon and chlorine) analysis of 1,1,1-TCA and 1,1-DCA biotransformation by an enriched microbial culture commonly used in field remediation. The isotopic fractionation (ε) values for carbon and chlorine are −5.8 ± 0.8‰ and −2.8 ± 0.3‰, respectively, for 1,1,1-TCA and are −8.9 ± 1.0‰ and −4.3 ± 0.5‰, respectively, for 1,1-DCA. Dual-isotope plots produced slopes (Λ_Cl/C_) of 2.1 ± 0.1 for 1,1,1-TCA and 2.1 ± 0.2 for 1,1-DCA, consistent with previously reported Λ_Cl/C_ values for abiotic transformation, despite significant differences in ε values, suggesting a masking effect that suppresses carbon and chlorine isotopic fractionation. Significantly, chlorine isotope values in the formed products are more enriched than the substrates, which may be diagnostic for masking effects in chlorinated organic compounds.

## Graphical abstract

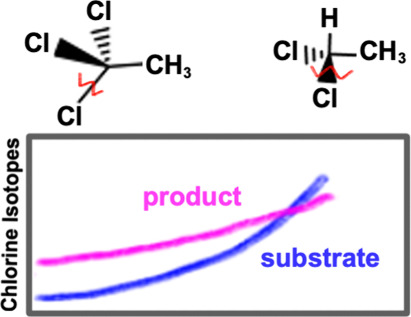

## Introduction

Chlorinated ethanes, including 1,1,1-trichloroethane (1,1,1-TCA) and 1,1-dichloroethane (1,1-DCA), are typically used as chemical precursors and were historically used as cleaning and degreasing solvents. Due to their adverse health and environmental effects, both compounds are listed as priority substances by the United States Environmental Protection Agency (U.S. EPA).^1,2^ Furthermore, 1,1,1-TCA is an ozone-depleting substance phased out by the Montreal Protocol.^3^ Both compounds are prevalent groundwater contaminants, present at up to a third of active National Priority List sites designated by the U.S. EPA (search of active NPL sites http://cumulis.epa.gov/supercpad/cursites/srchsites.cfm in April 2026). The long and pervasive legacy of these environmental contaminants means that multiple cleanup and remediation strategies are being developed and implemented around the world.

Biotransformation is generally more cost- and time-effective than abiotic remediation for many priority organic pollutants.^4,5^ For 1,1,1-TCA and 1,1-DCA, biotransformation has been investigated co-metabolically under oxic conditions^6^ and via reductive dehalogenation under anoxic conditions.^7^ In the latter case, specialized anaerobic microbes, such as *Dehalobacter* spp., use reductive dehalogenase (RDase) enzymes to gain energy by dechlorinating specific organohalides. Although complete biotransformation of chloroethane (CA) to ethane is theoretically possible, no experimental evidence currently supports this pathway.^7^

Compound-specific isotope analysis (CSIA) is a powerful tool to identify and calculate transformation rates at field sites by monitoring changes in contaminant isotopic compositions. Principles of CSIA and best practice guidance have been reviewed extensively elsewhere.^8^ Briefly, although molecules of the same compound have identical chemical compositions, differences in their isotopic compositions (isotopologues) can cause kinetic isotope effects (KIE). In many cases the magnitude of isotopic changes caused by physical processes (e.g., sorption, volatilization, diffusion) are typically smaller than from bond breakage in degradative reactions,^8^ allowing (bio)­chemical transformations to be distinguished from physical processes, particularly for carbon isotopes.^8^ For normal KIEs, molecules containing exclusively light isotopes of an element (^L^E) in the reactive position react slightly faster than those containing a heavy isotope (^H^E) at this position (primary) and/or its vicinity (secondary). These differences in reaction rates (^L^k vs ^H^k) lead to changes in isotopic compositions of reactant and product pools throughout the reaction (isotopic fractionation;^9^ commonly expressed as ε (see Materials and Methods)).

Using CSIA, the fraction of contaminant remaining is related to its isotopic composition using the Rayleigh model if a bulk isotopic fractionation, ε_E_,_bulk,_ is known, (ε_E_,_bulk_ ∝ 1/KIE).^10^ According to the Rayleigh model, in an irreversible, one-step reaction in a closed system with a normal KIE, the isotopic composition of a reaction product is more depleted in ^H^E than the reactant (e.g., see Figure 6 in ref 11). Hence, ε_E_,_bulk_ can be estimated from either substrate or product isotopic compositions although product isotope data are not always available in the literature, particularly for chlorinated ethanes.

Biotransformation reactions are comprised of multiple steps, with the overall net reaction typically referred to as reaction pathway.^12,13^ Each transformation step can proceed via different reaction mechanisms,^12,13^ i.e., variations in the order and manner of bond cleavage or formation.^14,15^ CSIA is particularly suited to evaluate so-called masking effects.^14^ Masking effects can arise from additional rate-limiting steps preceding the transformation step such as diffusion across cell membranes,^15–17^ nutrient limitations,^18^ mass transfer limitations,^15,16,19,20^ and enzyme–substrate binding.^14,21^ In such cases, while the fundamental intrinsic KIE associated with bond breakage is unchanged, the “observed” isotope effect (apparent kinetic isotope effect, AKIE) is smaller than the KIE when additional rate-limiting steps do not cause isotope fractionation.^22^ Comparing AKIE values for biotransformation with those produced in abiotic experiments, which are frequently used in the literature as a reference for fully expressed isotope fractionation (e.g., refs 14, 23, and 24) unless they are masked themselves, or theoretical calculations for bond breakage (e.g., using semiclassical Streitwieser limits^25^), can quantify the degree of masking, providing further insight into possible additional rate-limiting steps.

Previous studies of 1,1,1-TCA abiotic reductive dechlorination^26,27^ reported a range of carbon AKIE (AKIE_C_; 1.0158 to 1.030), occurring via concurrent hydrogenolysis and α-elimination pathways. These reactions are thought to proceed via an initial dissociative single electron transfer (SET) forming a common 1,1-dichloroethyl radical intermediate (Figure S1, Supporting Information section 1).^28,29^ Sherwood Lollar et al.^14^ is the only study to our knowledge that has investigated 1,1,1-TCA and 1,1-DCA biotransformation using CSIA. That study^14^ observed significant masking in whole-cell experiments using an enriched microbial culture (ACT-3; described in Materials and Methods) under anoxic conditions, with AKIE_C_ values of 1.0036 ± 0.0006 for 1,1,1-TCA and 1.0215 ± 0.013 for 1,1-DCA. Both observed fractionations were substantially smaller than the AKIE_C_ observed for abiotic transformation experiments (Figure S1A) and KIE estimates (1.03).^25^ Even greater masking was observed in cell-free extract experiments,^14^ ruling out membrane transport as the cause of masking. Instead, Sherwood Lollar et al.^14^ proposed that masking resulted from rate-limiting enzyme–substrate binding (commitment to catalysis).^30,31^

Dual-element isotope analysis is an established tool for biotransformation mechanistic investigations (recently reviewed by Ojeda et al.^13^) as single-element isotope analysis alone is often insufficient to investigate masking effects. Dual-element analysis (e.g., C,H or C,Cl) provides isotopic data for two different elements within a molecule, giving further insight into reaction mechanisms. Often, isotope data for the two elements produce a linear relationship with a slope (Λ) on a dual-isotope plot. This approach to dual-isotope plots notably assumes that (1 + *R*) ∼ 1 (a more thorough analysis is given in^32^), which chlorine isotopes do not satisfy due to the higher natural abundance of ^37^Cl. Nonetheless, mechanistic studies involving Λ rely on comparisons, and we remain consistent with existing literature for the most accurate comparisons. These Λ values are generally independent of masking effects since, if any additional rate-limiting step is not isotopically fractionating, the AKIE for both elements are masked to a similar degree, retaining their covariation. For example, Palau et al.^27^ used carbon and chlorine CSIA to investigate 1,1,1-TCA transformation by ZVI, reporting an AKIE_Cl_ of 1.0160 ± 0.006 and a Λ_C/Cl_ of 1.5 ± 0.1. They further demonstrated significantly different Λ_C/Cl_ among different transformation pathways (e.g., activated persulfate, hydrolysis/dehydrohalogenation), reflecting distinct underlying mechanisms, enabling the authors to differentiate among different abiotic transformation pathways.

This study is the first to our knowledge to investigate 1,1,1-TCA and 1,1-DCA biotransformation using the combined power of carbon and chlorine isotope effects to investigate large masking effects previously reported for these compounds. This study aims to apply dual (C, Cl) isotope analysis to a system where enzyme–substrate binding is hypothesized to be rate-limiting in the overall transformation pathway, reflected in Λ_C/Cl_. Furthermore, this work explores variations in reported isotopic fractionation across repeat experiments in the literature using the same system (ACT-3 TCA), to synthesize the knowns and remaining gaps in our collective knowledge of factors that control isotope fractionation in biotic systems.

## Materials and Methods

### Cultures and Growth Conditions

Biotransformation experiments were performed with the same enrichment culture (ACT-3) used by Sherwood Lollar et al.^14^ ACT-3 was originally derived from aquifer material collected at a 1,1,1-TCA-contaminated site in the northeastern United States.^15^ ACT-3 has been maintained for >20 years in mineral medium amended with 1,1,1-TCA and with an electron donor mixture of initially MEAL (methanol, ethanol, acetate, and lactate) and later EL (ethanol and lactate). The culture activity and microbial community have been stable over this time, particularly with respect to dechlorinating organisms (Figures S2 and S3).^33,34^ Some differences in the supporting members of the community may be due to changes in taxonomic classifications since 2010. ACT-3 contains *Candidatus* Dehalobacter alkaniphilus strain CF whose growth is linked to dechlorination of 1,1,1-TCA to 1,1-DCA, using the reductive dehalogenase CfrA, and *Ca*. D. alkaniphilus strain DCA, which dechlorinates 1,1-DCA to CA using reductive dehalogenase DcrA.^35^ Two separate subcultures were used in this study, both maintained with the EL mixture as electron donors: one fed with 1,1,1-TCA only (ACT-3 TCA/EL) and the other fed with 1,1-DCA only (ACT-3 DCA/EL).

### Microcosm Setup

Triplicate microcosms were prepared and sampled as described by Phillips et al.^36^ Each bottle was amended with a solution of HPLC grade ethanol and lactate (0.3 mL of a solution with 200 mM ethanol and 200 mM lactate; EL mixture) and either neat 1,1,1-TCA (54 μL) or 1,1-DCA (42 μL) to produce 1 mM aqueous concentration. Sterile controls containing 500 mL of autoclaved culture were prepared for each experiment. The 1,1,1-TCA used was an isotopically characterized in-house laboratory standard with a value of δ^13^C = −28.3 ± 0.4‰ expressed with respect to Vienna Pee-Dee-Belemnite (V-PDB).^8^ Unfortunately, no isotopically characterized in-house standard was available for added 1,1-DCA. Hence, the δ^13^C of the added 1,1-DCA was initially uncharacterized and fractionations are expressed versus a time zero instead of known δ^13^C. Both approaches are standard in such experiments.^8^ No chlorine isotope standards were available for either compound and hence these too are expressed versus a time zero. Bottles were capped with blue butyl stoppers (Bellco Glass Inc.) that were pretreated by boiling in 0.1 M solution of NaOH for 1 h and rinsed with deionized water.^37^ All bottles were stored on their side to minimize the likelihood of gas leakage. Fraction remaining values were corrected for dilution and mass removed (see Supporting Information section 4).^38,39^

### Analytical Methods

Detailed analytical methods are presented in the Supporting Information. Briefly, 1,1,1-TCA, 1,1-DCA, and CA concentrations were quantified via headspace analysis using a Hewlett-Packard 5890 series II gas chromatograph (GC) with a flame ionization detector (FID). Three-point calibration curves for 1,1,1-TCA, 1,1-DCA, and CA were checked daily. Reproducibility of daily standard measurements was used to quantify uncertainty, which was less than or equal to the typical uncertainty of GC/FID, ± 5%. Carbon isotope analysis was performed on a Thermo Scientific MAT 253 isotope-ratio mass spectrometer (IRMS) interfaced with an Agilent 7890 A GC system (Agilent) via a GC-IsoLink and a ConFlo IV (Thermo Scientific). Two separate mixtures of external reference materials (USGS 67, USGS 61, USGS 71) and in-house working standards (1,2-DCA, 1,1,2-TCA) were injected daily before and after sample runs to ensure measurement accuracy. Total δ^13^C uncertainty incorporating both accuracy and reproducibility is ±0.5‰.^40,41^ Chlorine isotope measurements were performed on a Neptune MC-ICPMS (Thermo Fisher Scientific, Germany) interfaced with a Thermo Scientific Trace 1310 GC, using previously established methods.^42,43^ Three offline characterized in-house standards (measured using direct inlet IRMS; 1 methyl chloride and two different trichloroethenes) were used to normalize sample measurements on the Standard Mean Ocean Chloride (SMOC) scale. The highest precision (1σ) observed for sample and control measurements was 0.3‰.

### Calculations

Bulk isotopic fractionation during contaminant transformation was assessed using CSIA. Isotopic ratios (*R*) are expressed in delta notation (δ, in ‰):

1 $$\delta^{H}E\left(‰\right)=\left(R_{\mathrm{sample}}/R_{\mathrm{standard}}\right)-1$$

where *R*_sample_ is the ratio of heavy to light isotope of the element of interest (e.g., ^13^C/^12^C or ^37^Cl/^35^Cl) in the sample, and *R*_standard_ is the corresponding isotopic ratio in the international reference standard (e.g., V-PDB for carbon and SMOC for chlorine).^8^

The evolution of the isotopic composition (δ^H^E) during transformation was further related to reaction extent using the Rayleigh model:

2 $$\ln\left[\left(\delta^{H}E_{t}+1\right)/\left(\delta^{H}E_{0}+1\right)\right]=\varepsilon_{E,\mathrm{bulk}}\cdot \ln f$$

where δ^H^E_t_ is the isotopic composition at time t, δ^H^E_0_ is the initial isotopic composition, ε_E_,_bulk_ is the isotopic fractionation in ‰, and f is the fraction remaining.

AKIEs were calculated from ε_E_,_bulk_ using eq 4:^44^

3 $$\mathrm{AKIE}=\frac{1}{1+\frac{n}{x\cdot z\left(\varepsilon_{E,\mathrm{bulk}}\right)}}$$

where *n* is the total number of atoms of the element of interest (e.g., C or Cl) in the molecule, *x* is the number of atoms in the reactive position, and *z* is the number of atoms in equivalent reactive positions. For AKIE_C_ calculations, *n* = 2, *x* = *z* = 1 for both 1,1,1-TCA and 1,1-DCA. For AKIE_Cl_ calculations, *n* = *z* = *x* = 3 for 1,1,1-TCA and *n* = *z* = *x* = 2 for 1,1-DCA as, for both compounds, all C–Cl bonds are equivalent and compete for reaction. Further details on calculations is presented in the Supporting Information.

## Results and Discussion

Sterile controls (SC) showed no detectable changes in mass (Figure 1A, B), δ^13^C, or δ^37^Cl values (data not shown) throughout the experiments. Standard deviations for SC replicates for the 1,1,1-TCA experiment (δ^13^C = −28.0 ± 0.1‰, δ^37^Cl = 1.4 ± 0.1‰), and for the 1,1-DCA experiment (δ^13^C = −32.0 ± 0.3‰, δ^37^Cl = −0.5 ± 0.2‰) were all well within the total analytical uncertainty for carbon (±0.5‰^8^) and chlorine (±0.6‰, based on 2σ standard deviations of standard measurements).

**Figure 1:**
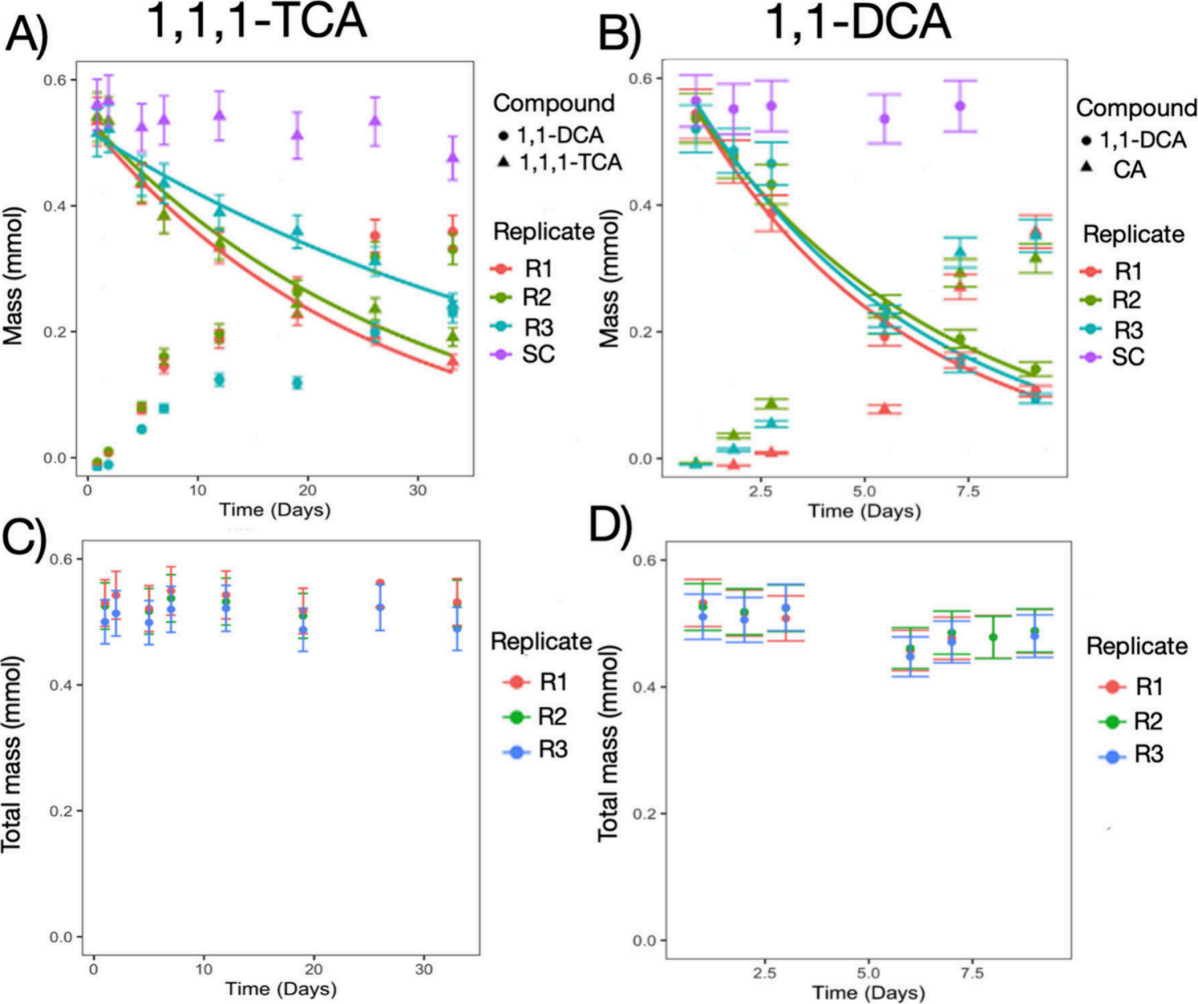
Mass vs time profiles for all replicates (R1–R3) and the sterile control (SC) of 1,1,1-TCA and 1,1-DCA in the ACT-3 TCA/EL experiment (A) and 1,1-DCA and CA in the ACT-3 1,1-DCA/EL experiment (B). First-order transformation rate lines are shown for each replicate. Total mass showing combined parent (circle) and daughter product (triangle) mass balance for 1,1,1-TCA experiment (C) and 1,1-DCA experiment (D). All mass values were calculated using the dimensionless gas–water partitioning constants for 1,1,1-TCA (0.7), 1,1-DCA (0.23), and CA (0.456) sourced from the U.S. EPA (https://www3.epa.gov/ceampubl/learn2model/part-two/onsite/esthenry.html). Error bars show analytical error propagated in the total mass calculation and show conservation of mass throughout the experiments, within error.

### Relationships between Isotope Fractionation and Transformation Pathways between 1,1,1-TCA and 1,1-DCA

Transformation curves for the conversion of 1,1,1-TCA to its product 1,1-DCA and 1,1-DCA to its product CA, together with the total mass balance of parent and daughter products are presented in Figure 1. Complete mass balances (Figure 1) were observed for both 1,1,1-TCA (Figure 1A,C) and 1,1-DCA (Figure 1B,D), consistent with a one-step dechlorination pathway (hydrogenolysis). ε_C,bulk_ and ε_Cl,bulk_ were calculated from Rayleigh plots (Figure 2) following eq 3, and the corresponding AKIE values were calculated using eq 4. Data showed good fit to the Rayleigh model, with R^2^ exceeding 0.9 in all cases (Figure 2).

**Figure 2:**
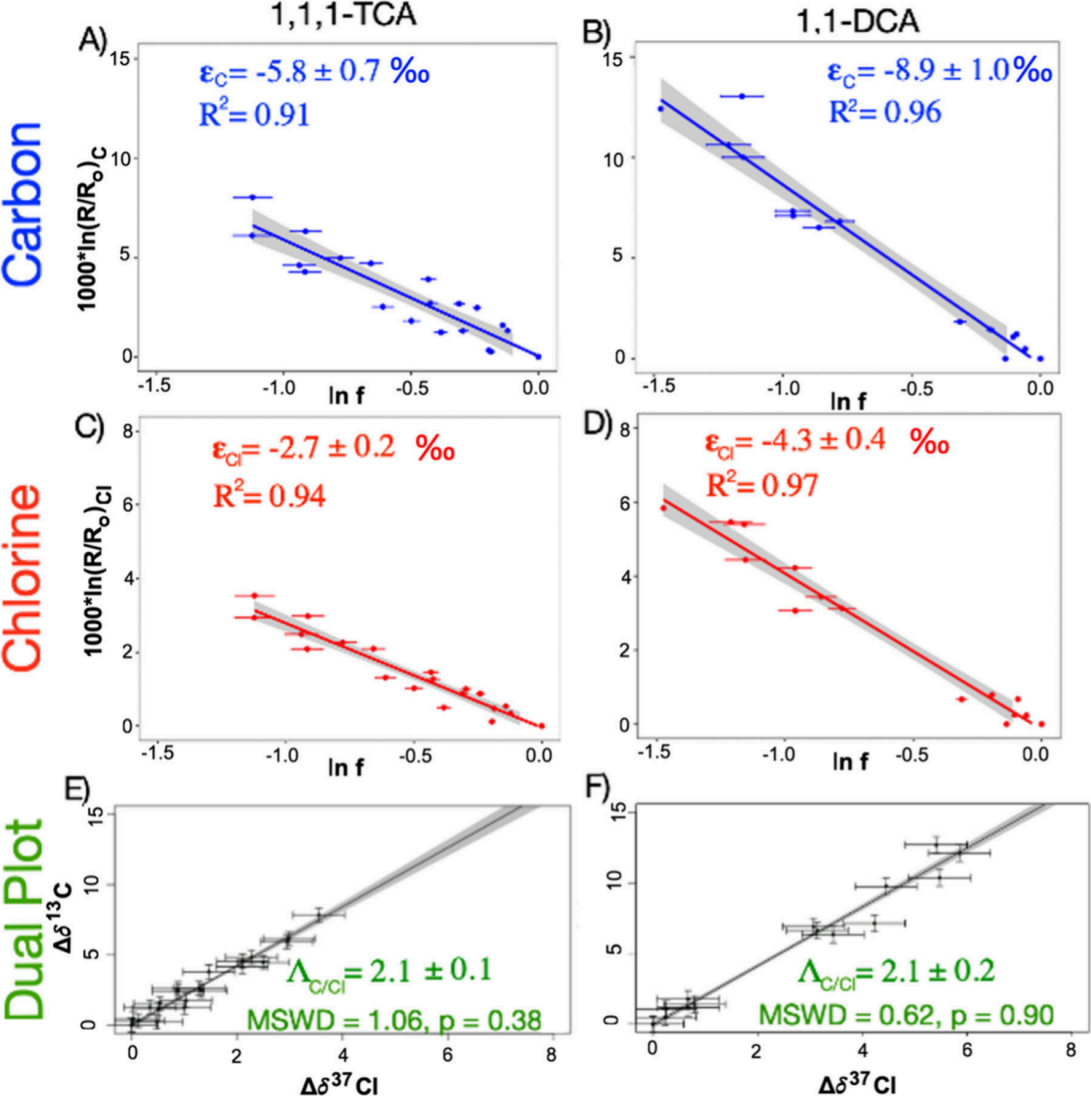
Rayleigh plots for calculating ε_C_ for 1,1,1-TCA (A) and 1,1-DCA (B) and ε_Cl_ for 1,1,1-TCA (C) and 1,1-DCA (D). Dual-isotope plots for determining Λ_C/Cl_ for 1,1,1-TCA (E) and 1,1-DCA (F). In Rayleigh plots, error bars represent propagated uncertainty of concentrations (*x*-axes) and isotope measurements (*y*-axes). Where not visible, error bars are smaller than plotted symbols. In dual-isotope plots, error bars reflect analytical uncertainty of each isotope system. Λ_C/Cl_ was calculated using the York regression method, following the recommendations by Ojeda et al.^60^ Associated output parameters, including mean square of weighted deviates (MSWD) and *p*-value, are shown in panels E and F. Gray shaded regions around the best fit lines indicate the calculated 95% confidence interval, representing uncertainty of ε and Λ.

1,1-DCA is transformed by ACT-3 DCA/EL via hydrogenolysis. Different reaction mechanisms have been proposed for hydrogenolysis, including direct interaction of the super reduced cobalt (Co^I^) and the substrate (e.g., S_N_1, S_N_2) and single electron transfer (SET) from the reduced cobalt leading to radical formation.^45^ Compared to 1,1,1-TCA, 1,1-DCA transformation has been sparsely investigated. The only reported AKIE_C_ for 1,1-DCA is the abiotic transformation by Zn^0^, proceeding primarily via α-elimination (95%) with minor hydrogenolysis component (5%).^46^ This complicates comparing with biotransformation studies where hydrogenolysis is the dominant pathway. However, both α-elimination and hydrogenolysis likely proceed via SET, allowing for tentative comparison of their respective AKIE_C_ values. Alternative reaction mechanisms for each pathway such as bimolecular nucleophilic substitution (S_N_2) are associated with large KIE_C_ values (1.03–1.09).^44^ A different reaction mechanism involving a unimolecular nucleophilic substitution (S_N_1) for 1,1-DCA transformation by ACT-3 DCA/EL cannot be ruled out as the measured AKIE_C_ of 1.0181 ± 0.0022 falls within the range expected for S_N_1 reactions (KIE_C_ = 1.0–1.03).^44^ However, electroparamagnetic resonance (EPR) studies on reductive dehalogenases (RDases) suggest that the cobalt in these enzyme exists in a super-reduced (Co^I^) state,^47,48^ and the high nucleophilicity of this state favors S_N_2-type pathways.^49^ Despite the possible mechanistic similarities (likely SET) between the abiotic and biotic transformation of 1,1-DCA, the abiotic AKIE_C_ for 1,1-DCA transformation by Zn^0^ (1.04–1.05) ^46^ remains significantly larger than the biotic AKIE_C_ reported here (1.0181 ± 0.0022). This discrepancy adds additional support that the carbon isotope effect for biotic 1,1-DCA transformation is masked, consistent with the earlier carbon isotope studies done on these compounds.^14^

Although both substrates undergo transformation via the same hydrogenolysis pathway, the observed AKIE_C_ values differed for the two compounds (1.0118 ± 0.0017 for 1,1,1-TCA and 1.0181 ± 0.0022 for 1,1-DCA; Table S2). Since in principle AKIE_C_ values should be comparable for different compounds transformed via the same reaction mechanism, with similar transition state structures and accounting for primary isotope effects only, this finding suggests differences in the mechanism and/or masking effects are present for these compounds.^50,51^ If masking effects alone are responsible for differences (due to additional rate-limiting steps that do not cause isotope fractionation), AKIE values should be suppressed to a similar degree for both elements, yet, while AKIE_C_ are different, the AKIE_Cl_ values were nearly identical for both compounds (1.0084 ± 0.0008 for 1,1,1-TCA and 1.0087 ± 0.0008 for 1,1-DCA; Table S2). However, comparing AKIE values between compounds is complicated, particularly as there are many factors (transition state structures, secondary isotope effects, masking effects) that may contribute to different observed AKIE between compounds. Semiclassical Streitwieser limits assuming 50% C–Cl cleavage in the transition state predict higher intrinsic carbon isotope effects (AKIE_C_ = 1.03, ε_C_,_bulk_ ∼ −15‰) but similar chlorine isotope effects (AKIE_Cl_ = 1.007, ε_Cl_,_bulk_ ∼ −2.1‰ for 1,1,1-TCA, and ∼ –3.3‰ for 1,1-DCA) compared to those observed. The lower AKIE_C_ value observed for 1,1,1-TCA compared to 1,1-DCA, despite similar AKIE_Cl_ values, is inconsistent with a shared nonfractionating rate-limiting step, which would be expected to suppress both isotope effects equally. Instead, the differences in AKIE_C_ values observed between 1,1,1-TCA and 1,1-DCA point to some fundamental difference in the transformation reaction or secondary isotope effects for these two compounds. Three possible scenarios include either different reaction mechanisms (as reported for chlorinated ethenes,^52^ where growth on different substrates affects RDase expression and thus reaction mechanism), reaction mechanisms that are impacted by different isotopically fractionating rate-limiting steps,^53,54^ or secondary isotope effects that impact the two compounds differently.

### Occurrence of Masking Effects

As discussed earlier, Palau et al.^27^ hypothesized that significant variability in AKIE_C_ values observed during abiotic reductive dehalogenation of 1,1,1-TCA may be due to masking effects, such as organohalide–metal complex formation. Even compared to this range, AKIE_C_ values for 1,1,1-TCA biotransformation observed in this study and in previous work^14^ are consistently lower, supporting the hypothesis that biotic transformation is also affected by a masking causing AKIE_C_ suppression, and the masking in biotic systems is larger than the organohalide–metal complexes involved in abiotic transformation.

To explain this suppression, Sherwood Lollar et al.^14^ proposed masking effects involved in biotransformation of 1,1,1-TCA and 1,1-DCA result from a well-known effect of multiple reaction steps referred to as commitment to catalysis (Scheme 1; eq 1 ^30,31^ ). Briefly, this model describes a two-step enzymatic reaction in which the enzyme (E) binds the substrate (S) to form an ES complex, followed by an irreversible step resulting in the formation of the product (P). The significant masking effects observed for both 1,1,1-TCA and 1,1-DCA biotransformation suggest that the first step (formation of the ES complex) created an additional rate-limiting step, thereby suppressing the AKIE_C_ of the bond breaking step (Scheme 1; eq 4):^30,31^

**Scheme 1:**
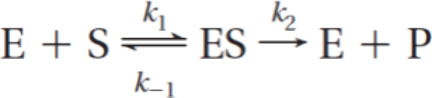


The relationship between the AKIE and KIE from commitment to catalysis is given by eq 4:

4 $$\mathrm{AKIE}=\frac{\mathrm{KIE}+C}{1+C},,\mathrm{where}C=k_{2}/k_{-1}$$

where *k*_2_ and k_–1_ are the rate constants shown in Scheme 1 for the transformation and dissociation step of the ES complex, respectively. Equation 1 shows that a highly efficient enzymatic reaction where *k*_2_ ≫ k_–1_, the value of C increases, causing a suppression of the AKIE relative to the intrinsic KIE (=^H^ *k*/^L^ *k*). Importantly, eq 1 assumes negligible fractionation associated with the formation and dissociation step of the ES complex (i.e., ^H^ *k*_1_ = ^L^ *k*_1_, ^H^ *k*_–1_ = ^L^ *k*_–1_). However, in cases where a fractionating step is present such as binding isotope effects (BIEs),^50^ its influence can be included in the expanded forms of eq 1 (e.g., ref 55 and refs therein). Although Sherwood Lollar et al.^14^ ruled out membrane transport as an additional rate-limiting step causing masking of the isotope effect (discussed above), the relevant masking effect could not be identified using carbon isotope CSIA alone. Sherwood Lollar hypothesized that 1,1,1-TCA and 1,1-DCA transformation was highly efficient (high *k*_2_ in Scheme 1), leading to rate-limiting enzyme substrate binding (eq 4). Different degrees of suppression in 1,1,1-TCA transformation by CfrA vs 1,1-DCA transformation by DcrA^14^ due to differences in enzymatic efficiency (more suppressed isotope effects in 1,1,1-TCA biotransformation; i.e. higher *k*_2_/k_–1_ than in 1,1-DCA biotransformation) was supported by different Michaelis–Menten constants (*K*_M_ = (*k*_–1_ + *k*_2_)/*k*_1_), concentration where rate is half of the maximum rate) values observed for each enzyme/substrate combination. A lower *K*_M_ was reported for 1,1,1-TCA in cell-free extracts (45 ± 18 μM) compared to 1,1-DCA (413 ± 64 μM), implying a higher affinity of CfrA to 1,1,1-TCA than of DcrA to 1,1-DCA.^39^ However, since *K*_M_ is a function of both forward (*k*_2_) and backward (*k*_–1_) reactions of enzyme–substrate complexation and transformation, differences in *K*_M_ alone cannot fully quantify the degree of isotope masking.

Results from the current study support that suppressed fractionation relative to abiotic reactions due to the efficiency of the enzyme-reaction pair involved. Taken together, these results are consistent with commitment to catalysis for 1,1,1-TCA transformation by CfrA and 1,1-DCA transformation by DcrA. The higher suppression observed for 1,1,1-TCA/CfrA suggests a higher commitment to catalysis (higher rate limitation) compared to 1,1-DCA/DcrA. However, we note that while these results are consistent with the commitment to catalysis hypothesis, our study does not confirm that enzyme substrate binding is indeed the rate-limiting step preceding C–Cl bond breakage; this step may involve electron transfer or other rate-limiting steps yet to be characterized.

Carbon and chlorine isotope effects for biotransformation reactions involving the mixed culture ACT-3 provide key comparisons for evaluating controls on ε_C_, ε_Cl_, Λ_Cl/C_. There are two characterized RDases (CfrA and DcrA) in ACT-3 with different substrate specificity,^56,57^ where the same RDase (CfrA) shows significant differences in isotope effects for different compounds. CF biotransformation by CfrA is associated with high carbon and chlorine isotope effects that are consistent with theoretical KIEs and transformation by vitamin B_12_ ^57^ (corrinoid cofactor within RDases). These results indicate a shared reaction mechanism between CfrA and vitamin B_12_ for CF biotransformation, which Heckel and Elsner^58^ identified as S_N_2. Masked carbon and chlorine isotope effects have been observed for CF biotransformation by other RDases that share high sequence similarity with CfrA.^36,57,59^ The “fully expressed” carbon and chlorine isotope effects for CF biotransformation by CfrA are in stark contrast to the likely masked isotope effects for biotransformation of 1,1,1-TCA by CfrA and 1,1-DCA by DcrA.

### Carbon Isotope Results across ACT-3 Culture Studies

A comparison between carbon isotope results from this work and previous ACT-3 experiments^14,61^ is summarized in Table S1. For 1,1-DCA, the AKIE_C_ (1.0181 ± 0.0022) is consistent with the results reported by Sherwood Lollar et al.^14^ in both whole-cell (1.0215 ± 0.0013) and cell-free extract (1.0161 ± 0.0019) experiments, suggesting that, as found in the earlier study, fractionation in this system is not impacted by substrate diffusion across the cell. These results demonstrate that the overall enzymatic reaction mechanism and kinetics for 1,1-DCA within the ACT-3 DCA/EL culture have remained stable over the past decade.

In contrast, significant differences in the AKIE_C_ were observed for 1,1,1-TCA transformation when compared to results from Sherwood Lollar et al.^14^ (Table S1). Two hypotheses could account for these differences in ε_C,bulk_ for 1,1,1-TCA transformation by ACT-3 TCA/EL: (1) differences in substrate bioavailability that limit mass transfer and consequently the reaction order of enzyme–substrate binding^19,20,62^ (Figure S3) or (2) an amino acid mutation in CfrA that may affect enzyme–substrate binding efficiency or transition state stabilization (Figure S4). Both hypotheses are described in detail in the Supporting Information (section 5). Recent work highlighted the importance of specific amino acids in CfrA for substrate specificity and mechanism,^63^ and amino acid mutations would likely impact isotope fractionation of the same substrate^36^ Notably, either scenario remains consistent with a masking system where observed isotope fractionation is dependent on the relative rate constants for transformation and substrate dissociation (eq 1 and Scheme 1), which continues to provide an accurate framework for interpreting isotope fractionation for both 1,1,1-TCA and 1,1-DCA experiments (with the potential involvement of an interplay between mass transfer and enzyme substrate binding in hypothesis 1). Hence, while there are different potential causes for differences in the AKIE_C_ observed for 1,1,1-TCA transformation by ACT-3, all are consistent with changes in enzyme–substrate binding causing a different degree of masking of the carbon isotope effect, although these results cannot definitively confirm masking is due to commitment to catalysis.

### Presence of a Nonfractionating Rate-Limiting Step

Interpreting different reaction mechanisms or secondary isotope effects cannot be based on single element isotope effects (AKIE) alone. Further investigation of this hypothesis by dual-element isotope analysis for 1,1,1-TCA and 1,1-DCA had not been attempted until now. Λ_Cl/C_ for 1,1,1-TCA and 1,1-DCA transformation are given in Figure 2. The output parameters mean square of weighted deviations (MSWD) and associated p-values indicate good fit to the York regression method.^62^ To the best of our knowledge, no prior studies have reported Λ_Cl/C_ for 1,1,1-TCA for biotic transformation, nor for 1,1-DCA for either biotic or abiotic transformation. Λ_Cl/C_ for 1,1,1-TCA transformation by ACT-3 TCA/EL observed here (2.1 ± 0.1) is similar to, but statistically different from, a previously reported Λ_Cl/C_ for abiotic transformation by ZVI (1.5 ± 0.1)^27^ (Figure 3C), with a hypothesized SET mechanism. While these values fall outside each other’s uncertainty range, the scarcity of reported Λ_Cl/C_ values for 1,1,1-TCA makes a meaningful mechanistic comparison challenging. For context, Λ_Cl/C_ reported for perchloroethene (PCE) transformation (ranging between 3.7 and 5.3) have all been attributed to the same underlying reaction mechanism although the specific values did not agree within uncertainty.^52^ This suggests that small differences in Λ_Cl/C_ do not necessarily indicate different mechanisms. Reported Λ_Cl/C_ can be difficult to compare as uncertainties have been traditionally underestimated, leading to interpretations of different mechanisms for Λ_Cl/C_ that in fact are a result of the same underlying mechanism.^60,64^ Additional dual-isotope studies assessing carbon and chlorine isotope fractionation for chlorinated ethane transformation may help to address this issue, using the York method for regression of dual-isotope plots to calculate Λ_Cl/C_ to accurately incorporate all uncertainty in the calculation.^60^ A community-wide robust development of frameworks to report uncertainties is a critical step forward in resolving dual-element isotope interpretations.^60,64,65^ Importantly, the study of Palau et al.^27^ predates these recommendations and used ordinary least-squares regression to calculate Λ_Cl/C,_ likely resulting in an underestimate of total uncertainty. Overall, the subtle difference (0.6) between the Λ_Cl/C_ value reported here (2.1 ± 0.1) and for ZVI transformation (1.5 ± 0.1)^27^ suggest that 1,1,1-TCA biotransformation by ACT-3 and transformation by ZVI may proceed via a similar reaction mechanism. Interestingly, the parameters calculated in this study (Figure 2) are very similar to a previous study^66^ investigating 1,1,2-TCA biotransformation by a *Dehalogenimonas sp*.-containing mixed culture (ε_C_,_bulk_ = −6.9 ± 0.4, ε_Cl_,_bulk_ = −2.7 ± 0.3, Λ_Cl/C_ = 2.5 ± 0.2) where the reaction pathway was dichloroelimination and masking effects were proposed. Although the reaction pathways between these studies are different (dichloroelimination vs hydrogenolysis), they may share a common reaction mechanism (e.g., SET).

**Figure 3:**
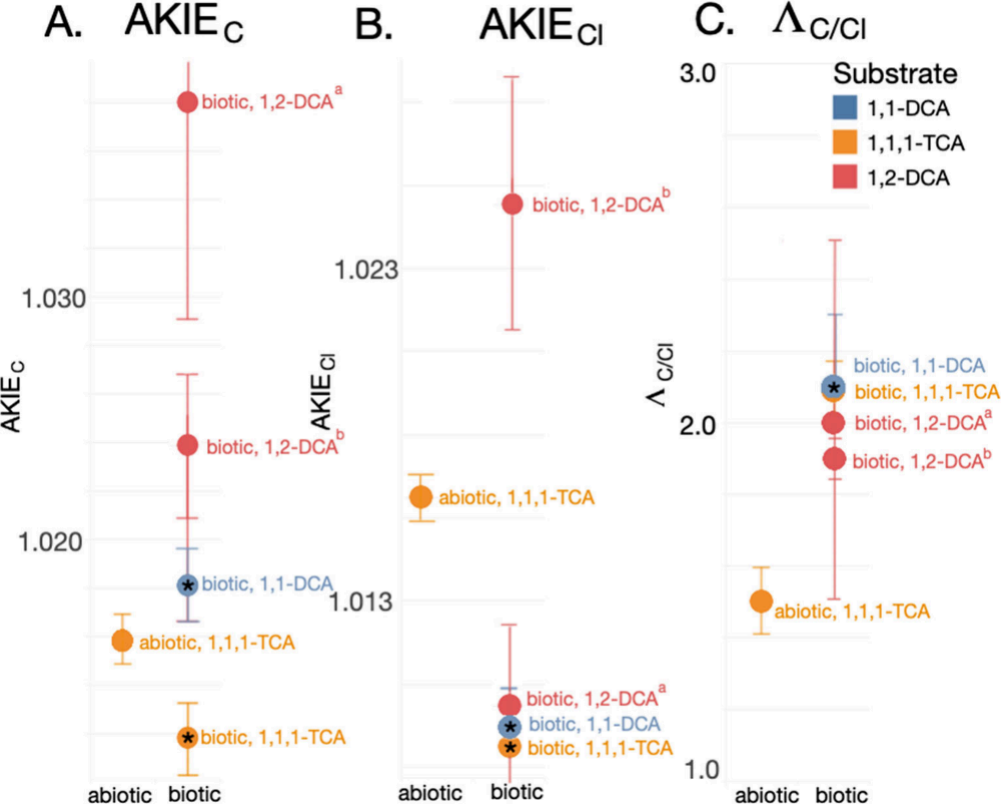
Comparison of AKIE_C_ (A), AKIE_Cl_ (B), and Λ_C/Cl_ (C) values for abiotic and biotic transformation of chlorinated ethanes, as indicated on the *x*-axes. Data include results for 1,1-DCA (blue; this study), 1,1,1-TCA (orange; this study and Palau et al.^27^), and 1,2-DCA (red; Franke et al.^67^ (indicated by ^a^) and Dhg from Palau et al.^24^ (indicated by ^b^)). Data from this study are marked with “*”. Error bars represent error in AKIE values (calculated from ε and propagated through eq 4). Refer to the main text for detailed discussion of observed trends and mechanistic implications.

The closest available dual-isotope studies for comparison with 1,1-DCA biotransformation are Franke et al.^67^ and Palau et al.;^24^ both examined 1,2-DCA biotransformation. Franke et al.^67^ used *Dehalococcoides mccartyi* strain BTF08 expressing the RDase VcrA. They^67^ reported a Λ_Cl/C_ value of 2.0 ± 0.5, very similar to the value for 1,1-DCA transformation observed in this work (2.1 ± 0.2). The authors listed several possible reaction mechanisms, including SET (inner or outer sphere) and cobalt-centered nucleophilic attack of the halogen with concomitant removal of vicinyl halogens, but did not definitively suggest either mechanism for 1,2-DCA biotransformation. Palau et al.^24^ investigated 1,2-DCA biotransformation by Dehalococcoides (Dhc)- and Dehalogenimonas (Dhg)-containing cultures, with Λ_Cl/C_ of 6.8 ± 0.2 and 1.89 ± 0.02, respectively, corresponding to different reaction mechanisms. Heckel et al.^58^ later classified these reactions as S_N_2 (Dhc) and a separate, non- S_N_2 reaction (Dhg). The Dhg culture is included in Figure 3 for comparison, while the Dhc culture is not due to a likely different mechanism.

The agreement of Λ_Cl/C_ values between abiotic and biotic transformation, despite significantly different AKIE_C_ and AKIE_Cl_ reported by this study and previous work (Figure 3) suggests there is a common mechanism underlying both processes for both compounds and the elemental fractionation therefore covaries^27,67^ (as described above). This consistency supports the interpretation that the observed suppression of AKIE_C_ for 1,1,1-TCA and 1,1-DCA, relative to previous experiments,^27,67^ and to theoretical AKIE, are not due to a fundamentally different mechanism, but rather reflect the presence of an additional rate-limiting step that is nonfractionating in biotic vs abiotic transformation (Figure 3). Such a step can be caused by the initial enzyme–substrate binding.^30,31^ A schematic overview of the proposed transformation pathways for TCA and DCA transformation is given in the Supporting Information (Figure S5). However, as discussed earlier, the mechanism for 1,1,1-TCA transformation may be different from 1,1-DCA biotransformation. Differences could arise from the mode of SET (i.e., inner-sphere vs outer-sphere) or from how Co^I^ interacts with the substrate (e.g., SET vs Co attack of halogen). Additionally, the different trends between AKIE_C_ and AKIE_Cl_ may partly reflect different contributions of secondary chlorine isotope effects, influenced by the different number of chlorine atoms in 1,1,1-TCA and 1,1-DCA.

### Chlorine Isotopes

A unique aspect of this study is the product chlorine isotope compositions. For both the 1,1,1-TCA and 1,1-DCA experiments (producing 1,1-DCA and CA, respectively), the δ^13^C values of the products follow the Rayleigh model for a closed system, unidirectional reaction with a normal KIE (i.e., product compounds are more depleted in ^13^C than parent compounds, Figure 4A,B). In contrast, δ^37^Cl values for the products deviate markedly from this pattern. Rather than being depleted, the products are more enriched in δ^37^Cl relative to the parent compounds (Figure 4C,D). To the best of our knowledge, this phenomenon has only been reported by Badin et al.,^68^ who modeled the effect using secondary chlorine isotope effects. Despite this enrichment, the calculated ε_Cl_ are consistent between parent and product isotope data within uncertainty (Figure 4C,D). Analysis of product isotope compositions from a previous study^36^ of chloroform (CF) biotransformation by SC05-UT (AKA KB-1 Plus CF; Figure S7) reveals similar δ^37^Cl trends in the products for SC05-UT which also exhibited suppressed carbon and chlorine isotope effects. Interestingly, no such enrichment was observed for CF transformation by another microbial culture (ACT-3-CF) where both carbon and chlorine isotope effects were “fully expressed” (Figure S7). While enrichment of product relative to parent isotope signatures can result from simultaneous product formation and transformation,^69,70^ this is inconsistent with the data presented here since both experiments showed closed mass balances (i.e., consistent total mass accounting for both substrate and product; Figure 1C,D), making this scenario unlikely. Furthermore, if these trends were due to simultaneous product formation and transformation, similar enrichment trends would be expected in δ^13^C. Instead, the δ^13^C follow a trend characteristic of a single transformation step (i.e., production without simultaneous transformation; Figure 4A,B), further supporting the observed δ^37^Cl enrichment in products is not due to concurrent production and transformation processes.

**Figure 4:**
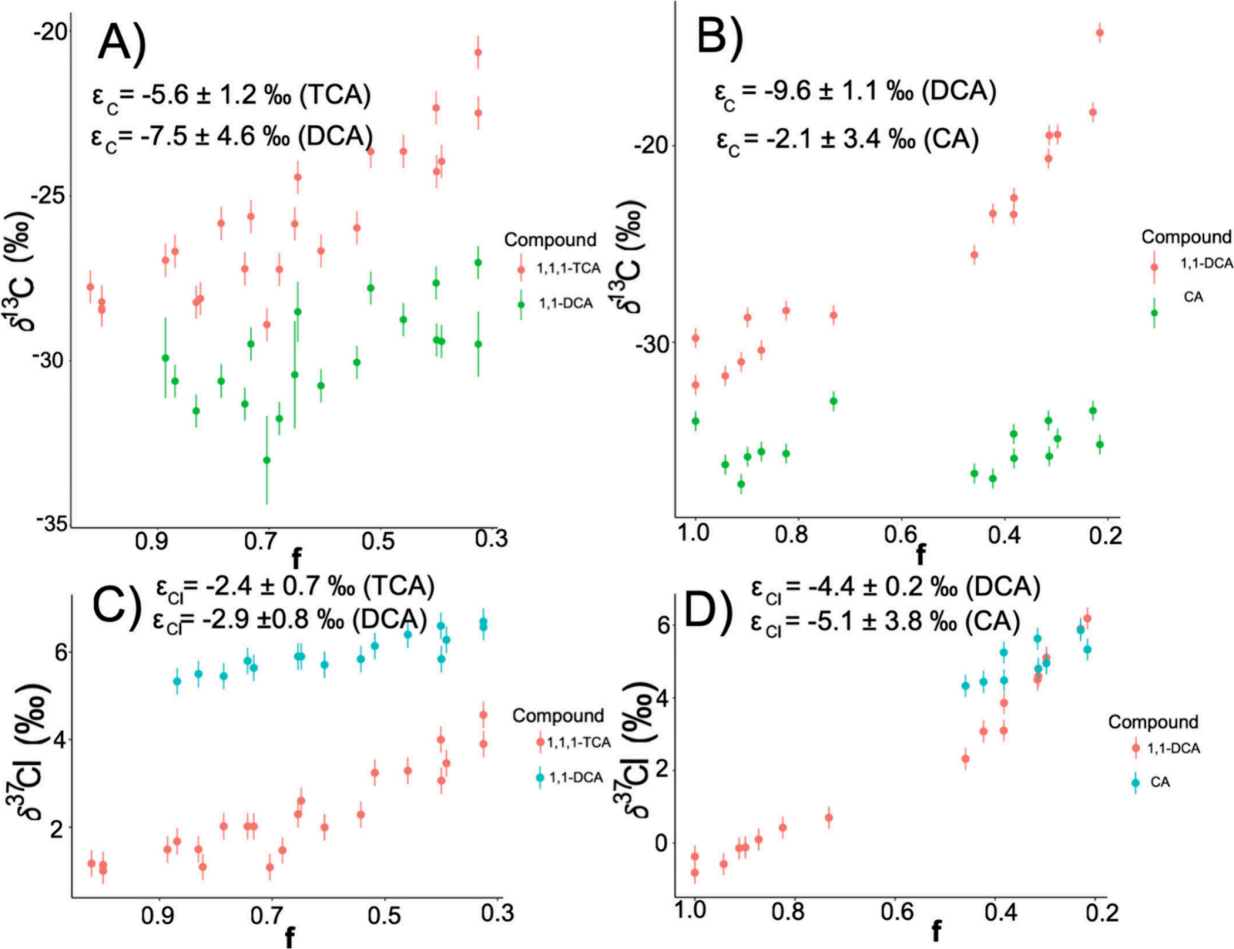
Rayleigh plots showing isotope fractionation for parent and daughter product for δ^13^C of 1,1,1-TCA transformation to 1,1-DCA (A), δ^13^C of 1,1-DCA transformation to CA (B), δ^37^Cl of 1,1,1-TCA transformation to 1,1-DCA (C), δ^37^Cl of 1,1-DCA transformation to CA (D). Error bars represent analytical uncertainty for each isotope system, based on sample and standard reproducibility. The ε_E_ values were calculated from parent or product isotope data using the Rayleigh model equations described by Cretnik et al., 2014. Brackets indicate the compound used to estimate ε_E_.

Although the mechanism underlying the δ^37^Cl remains unresolved, there are several possible explanations.^64,65^ Secondary isotope effects are a likely contributor,^71^ particularly as polychlorinated compounds are more prone to secondary chlorine isotope effects.^13,71^ Following the approach of Cretnik et al.,^71^ who show a comprehensive mathematical basis for secondary Cl isotope effects, both primary (ε_primary_) and secondary (ε_secondary_) Cl isotope effects were calculated for 1,1,1-TCA and 1,1-DCA transformation (Table S3). While these estimates are associated with relatively large uncertainty, positive ε_secondary_ values for both 1,1,1-TCA and 1,1-DCA transformation could cause observed product δ^37^Cl trends (Figure 4). We conceptualize this result as preferential C–Cl cleavage of molecules with ^35^Cl in the reactive position (lost) and ^37^Cl in a position in the vicinity (retained), leading to an enrichment of molecules retaining ^37^Cl in one of the retained Cl atoms in the product. While secondary isotope effects cannot be directly measured in this study, the calculated values for ε_secondary_ (Table S3) and their consistency with product δ^37^Cl (Figure 4) suggest they are present. Nonetheless, more explicit evidence is required to strengthen and validate this interpretation and fully resolve the observed isotope fractionation.

In addition, binding isotope effects (BIE; reviewed by Stratton et al.^50^) may also contribute to suppressed isotope effects in the substrate. Specifically, positive chlorine BIEs may suppress the parent isotope effect while resulting in an enrichment of the product in ^37^Cl. Conceptually, this equilibrium effect would involve an “ES” pool enriched in ^37^Cl relative to the bulk substrate, where the “ES” pool participates in the transformation step (Scheme 1). Therefore, while the KIE acts on ES → EP, a positive BIE makes it less likely that ^37^Cl is ‘returned’ to the substrate pool, creating a net effect of enriched product δ^37^Cl relative to substrate. While positive BIEs can explain the pattern of substrate and product δ^37^C, this scenario conflicts with Λ_C/Cl_; the consistent Λ_C/Cl_ values observed support the interpretation that the rate-limiting step, which may be enzyme–substrate binding, is nonfractionating with respect to C and Cl isotopes (discussed in previous section; Figure 4), suggesting BIEs are not present. Nevertheless, further measurements of AKIE_C_, ensure AKIE_Cl_ and Λ_C/Cl_ for both biotic and abiotic transformation of 1,1,1-TCA and 1,1-DCA are required to differentiate between masked and unmasked isotope effect scenarios more definitively.

While the origin of the δ^37^Cl enrichment in products of 1,1,1-TCA and 1,1-DCA biotransformation by ACT-3, and CF biotransformation by SC05-UT remains unknown, this trend consistently appears in systems where isotope masking effects due to enzyme–substrate binding has been proposed. Although further work, including a comprehensive mechanistic framework, is required to corroborate and validate this hypothesis, our findings suggest that product δ^37^Cl compositions may serve as a useful diagnostic indicator of masked isotope effects, specifically those arising from rate-limiting binding steps in enzymatic pathways.

The elevated chlorine isotope effects observed here may therefore reflect a combination of significant primary and secondary isotope effects acting in concert, as has also been suggested for abiotic 1,1,1-TCA transformation,^27^ 1,2-DCA biotransformation,^72^ and for biotransformation of chlorinated ethenes.^71^ Future investigation into secondary isotope effects is needed as their influence adds complexity to attempts to relate measured carbon and chlorine isotope effects to reaction mechanisms.

### Environmental Significance and Implications for Field Investigations

While CSIA is a powerful tool for assessing contaminant transformation in the field, estimates of ε_E_,_bulk_ and Λ are needed to apply CSIA for quantitative estimates of biotransformation extent in field applications, and to develop the most robust estimates of potential cleanup rates. As a means of overcoming masking effects, Λ can be used to identify the relevant transformation mechanism, and thereby select the most accurate estimates of ε_E_,_bulk_ to apply for estimating rates and extent of transformation.^13^ New statistical approaches provide more robust constraints on application of observed ε_E_,_bulk_ and Λ values,^60,64^ building on the previous best practice guidance from the EPA/IAEA regarding using the minimum, mean, and maximum values or bracket estimates of biotransformation performance.^8^ Further progress requires continuing to understand the fundamental factors that influence isotopic fractionation, for both single- and dual-element applications, particularly the role of rate-limiting steps. This work describes a system where enzymatic efficiency and multiple rate-limiting steps likely impact carbon and chlorine isotope fractionation during 1,1,1-TCA and 1,1-DCA biotransformation. New dual-element isotope data support the hypothesis that a nonfractionating enzyme–substrate binding step causes significant masking of observed fractionations relative to theoretically predicted KIE. Furthermore, this work highlights the potential for product δ^37^Cl compositions as an additional indicator for identifying masking effects.

## Supplementary Material



## Notes

Description of AKIE comparisons between biotic and abiotic studies of 1,1,1-TCA transformation with figure, ACT-3 community composition, detailed analytical methods, comparison of carbon isotopes between studies and discussion of differences, calculated AKIE_C_ and AKIE_Cl_, schematic of overall proposed hydrogenolysis pathway, δ^37^Cl for CF biotransformation by ACT-3 and SC05, and table showing estimated ε_primary_ and ε_secondary_ (PDF)
