# Supplementary material for: Isotopic Fractionation and Masking Effects during Biotransformation of Chlorinated Ethanes
Source: Environ Sci Technol. 2026 May 28;60(22):15940–51. doi: 10.1021/acs.est.5c14089 (PMC13261873; doi:10.1021/acs.est.5c14089)
Supplement: Supplementary file 1 [file es5c14089_si_001.pdf]

Supporting Information

Isotopic Fractionation and Masking Effects during Biotransformation of Chlorinated Ethanes

Elizabeth Phillips[†||]*, Joan De Vera*[†]*, Steffen Kümmel*[‡]*, Olivia Bulka*[§]*, Weibin Chen*[†]*, Elizabeth Edwards*[§]*, Ivonne Nijenhuis*[‡]*, Matthias Gehre*[‡]*, Barbara Sherwood Lollar*[†#*]

[†] Department of Earth Sciences, University of Toronto, 22 Ursula Franklin Street, Toronto, Ontario M5S 3B1, Canada

[||]The Grantham Institute for Climate Change and the Environment, Imperial College London, Exhibition Road, South Kensington, SW7 2AZ, UK

[§]Department of Chemical Engineering and Applied Chemistry, University of Toronto, 200 College Street, Toronto, Ontario M5S 3E5, Canada

[‡]Department of Isotope Biogeochemistry, Helmholtz Centre for Environmental Research – UFZ, Permoserstrasse 15, 04318 Leipzig, Germany

[#]Institut de Physique du Globe de Paris (IPGP), Université Paris Cité, 1 rue Jussieu 75238 Paris Cedex 05

Containing 22 pages including 7 figures (Figures S1-S6), 3 tables (Tables S1-S3), and 1 equation (Equation S1)

Description of AKIE comparisons between biotic and abiotic studies of 1,1,1-TCA transformation with figure, ACT-3 community composition, detailed analytical methods, comparison of carbon isotopes between studies and discussion of differences, calculated $AKIE_C$

and $AKIE_{Cl}$, schematic of overall proposed hydrogenolysis pathway, $\delta^{37}Cl$ for CF biotransformation by ACT-3 and SC05, and table showing estimated $\varepsilon_{primary}$ and $\varepsilon_{secondary}$.

* Corresponding author: Elizabeth.phillips@imperial.ac.uk

1. Comparing $AKIE_C$ for 1,1,1-TCA abiotic vs. biotic transformation

Figure S1A shows published carbon CSIA results (ε_C) for abiotic and biotic transformation of 1,1,1-TCA, with proposed mechanisms in Figure S1B. Previous studies of 1,1,1-TCA reductive dechlorination by ZVI[1,2] and FeS (the latter formed by biotically-mediated processes)[3] show carbon AKIE ranging from 1.0158 to 1.030. 1,1,1-TCA transformation by ZVI occurs via concurrent hydrogenolysis and α-elimination, with a proposed mechanism involving an initial dissociative single electron transfer (SET) forming a common 1,1-dichloroethyl radical intermediate.[4,5] Pathway-specific ε_C values estimated using the method described by Cretnik et al.[6] are included in Figure S1A as indicated by '*' (additional detail in SI section 2) In other experiments with ZVI, Elsner et al.[1] observed the same products during 1,1,1-TCA transformation with different ε_C values (-13.7 to -15.8‰), indicating a range in ε_C for abiotic 1,1,1-TCA transformation (Figure S1A) despite similar transformation pathways. Although hydrogenolysis-specific ε_C estimates are not possible for the ZVI data in Elsner et al.[1] or FeS data in Broholm et al. [3] (SI section 2) the common initial dissociative SET mechanism underlying the two pathways (see Figure S1B) suggests that similar ε_C values should be observed. Even considering this variation, the range of ε_C values observed for abiotic 1,1,1-TCA transformation is significantly larger than values observed for biotransformation of 1,1,1-TCA by ACT-3 TCA in whole cells and cell-free extracts[7] (Figure S1A).

2. Estimating pathway-specific ε_C values

Pathway-specific ε_C values can be estimated using the different intercepts observed for the data on a plot of $\delta^{13}C$ vs. fraction remaining (f), as described by Cretnik et al.[6] to tease apart the ε_C value for abiotic hydrogenolysis from α-elimination. Using the method outlined by Cretnik et al.[6],

the difference between the initial $\delta^{13}C$ of 1,1,1-TCA ($\delta^{13}C_{o,\ 1,1,1\text{-TCA}}$; -26.3 ± 0.1‰) and estimated intercept of the initial formed 1,1-DCA ($\delta^{13}C_{o,\ 1,1\text{-DCA}}$ ~-38‰, estimated from Figure 2E in Palau et al.[2]), the estimated ε_C for abiotic hydrogenolysis ($\varepsilon_C \sim \delta^{13}C_{o,\ 1,1,1\text{-TCA}} - \delta^{13}C_{o,\ 1,1\text{-DCA}}$) is ~ -11.7‰. This estimate is indicated by a '*' in Figure S1A.

A similar analysis was not undertaken for other abiotic data included in Figure 1 for abiotic 1,1,1-TCA transformation by ZVI[1] (daughter products were not quantified) or by FeS [3] (no $\delta^{13}C$ vs. f plots were provided in the publication).

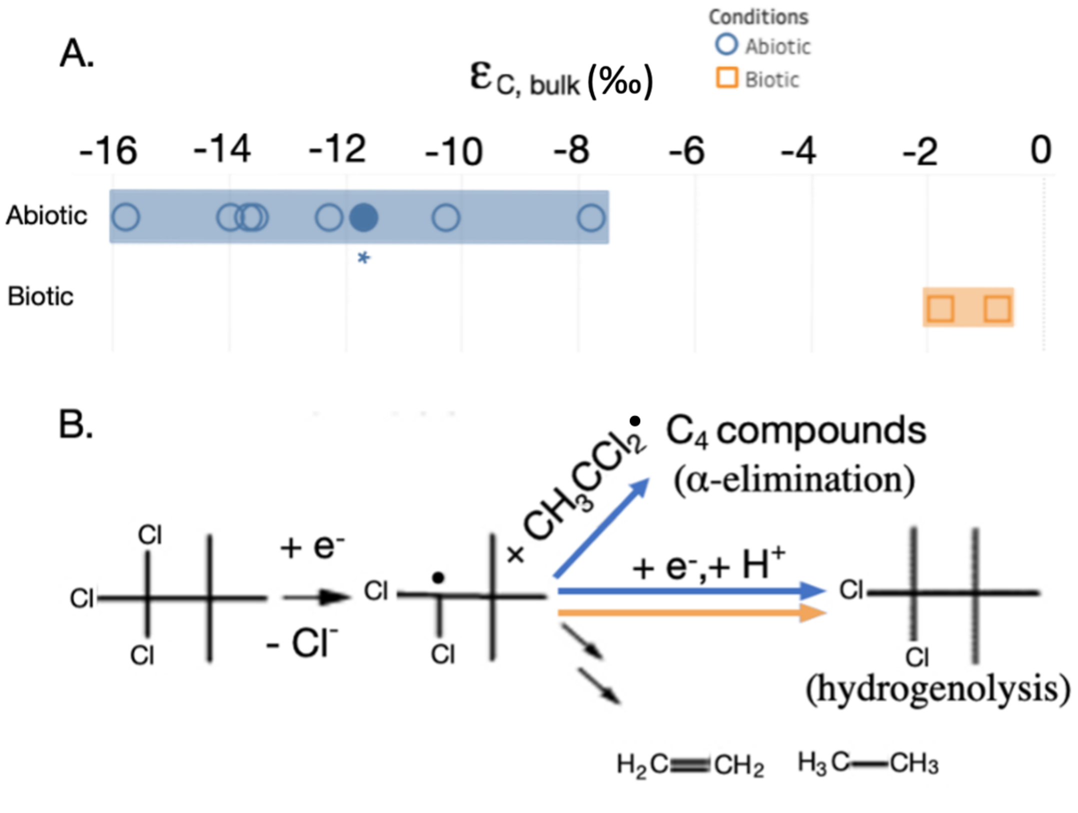

Figure S1. ε_C values in the literature[1–3] for abiotic (blue circles) and biotic (orange squares) transformation of 1,1,1-TCA (A). The filled in symbol with '*' within abiotic transformation

shows the estimated ε_C value from Palau et al.[2] (see text). A scheme of reaction mechanisms modified from Palau et al.[2] is shown in (B) for the two major pathways of 1,1,1-TCA reductive dechlorination: α-elimination (abiotic only) and hydrogenolysis (abiotic and biotic). Coloured arrows show the pathways for abiotic (blue) and biotic (orange). Both pathways involve an initial dissociative single electron transfer (SET) followed by additional steps that differentiate the pathways.

3. ACT-3 Culture Community Composition

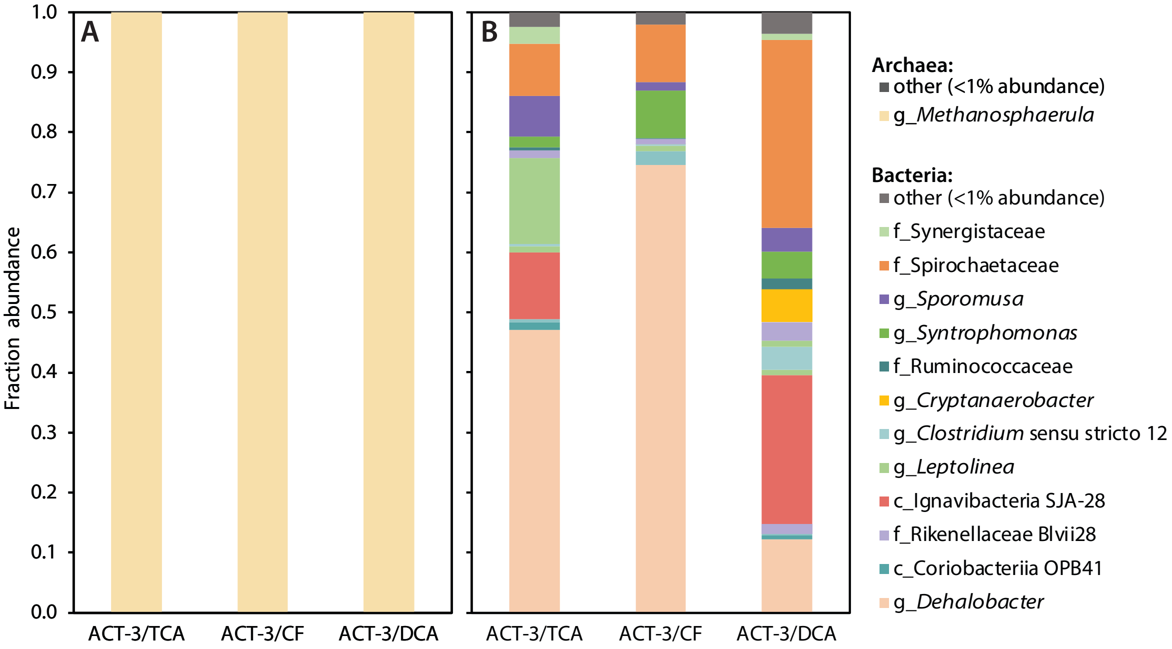

Figure S2. Community composition of ACT-3 subcultures in September 2019. Archaea (A) and bacteria (B) visualized separately to account for kingdom-specific sequencing biases. Each colour represents one amplicon sequence variant.

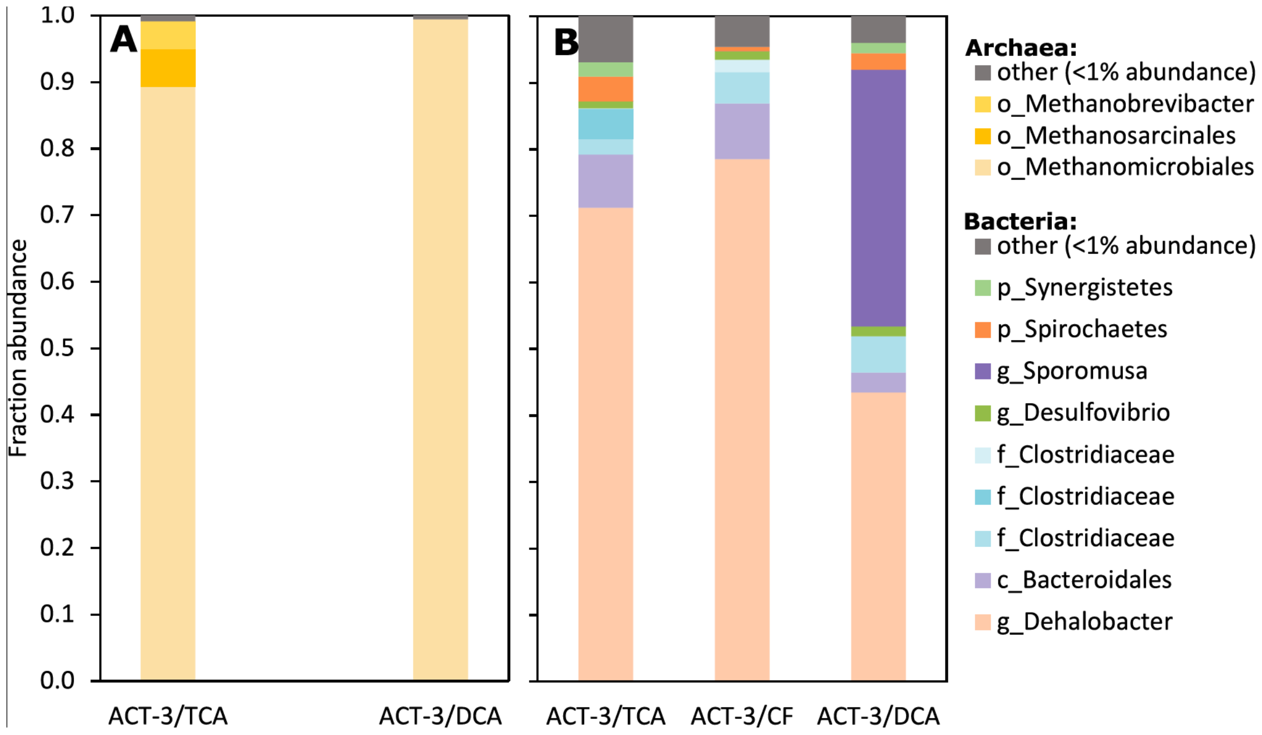

Figure S3. Community composition of ACT-3 subcultures from 16S pyrotag sequencing in 2010 (cite:10.1371/journal.pone.0052038). Archaea (A) and bacteria (B) visualized separately to account for kingdom-specific sequencing biases. Each colour represents one cluster as defined by pyro-clust (PyroTagger_TOJ_2010.pdf), performed by the Joint Genome Institute.

4. Analytical Methods

Concentration Measurements

1,1,1-TCA, 1,1-DCA, and CA concentrations were quantified via headspace analysis using a Hewlett-Packard 5890 series II GC-FID with an Agilent GSQ column (30 m x 0.53 mm), operated in splitless mode with a 250°C inlet temperature and 210°C detector temperature. The GC oven temperature program started at 50°C and was ramped to 155°C at 30°C min^{-1}, and then from 155°C to 180°C at 4°C min^{-1}. Helium was used as a carrier gas with a flow rate of 2 mL x min^{-1}. Headspace samples (0.3 mL) were withdrawn using a VICI Pressure-Lok gas tight syringe (Supelco). Three-point calibration curves of a standard mixture containing 1,1,1-TCA, 1,1-DCA,

and CA were prepared prior to experiments and standards were injected daily to ensure accuracy of the calibration and measure reproducibility. Relative standard deviations for samples and standards using this method were ± 5% based on standard error of the GC measurements.

Stable Carbon Isotope Analysis.

Prior to analysis, 1-3 mL was taken from the sample vials and added to a 5-mL crimp vial, shaken for several minutes, and incubated overnight at room temperature. Samples were analyzed from the headspace of crimp vials using a pressure lock gas tight syringe (Hamilton). Carbon isotope analysis was performed on a Thermo Scientific MAT 253 isotope-ratio mass spectrometer (IRMS) interfaced with an Agilent 7890 A GC system (Agilent) via a GC-IsoLink and a ConFlo IV interface (Thermo Scientific). The combustion reactor was maintained at 1000°C. Sample separation was done with a ZB-1 column (Phenomenex, 60 m x 0.32 mm x 1 µm film) held isothermally at 80°C and using a helium carrier gas flow of 2 mL min^{-1}. The injector temperature was kept at 250°C and a split ratio of 1:20, 1:5, or 1:3 was used depending on the concentrations of the samples. A mixture of external reference materials (USGS 67 ($\delta^{13}C_{defined}$ = -34.50‰), USGS 61 ($\delta^{13}C_{defined}$ = -35.10‰), USGS 71 ($\delta^{13}C_{defined}$ = -10.50‰)) and a mixture of in-house working standards containing 1,2-DCA (-28.9 ± 0.3‰) and 1,1,2-TCA (-10.1 ± 0.4‰) were injected daily before and after sample measurements to ensure measurement accuracy. A two-point calibration using external reference materials was applied to measured $\delta^{13}C$. The 1,2-DCA and 1,1,2-TCA standards were prepared and injected in the same manner as samples (1 mL in 5 mL crimp sealed cap vials) to test that the sampling procedure did not significantly affect $\delta^{13}C$. Total uncertainty of $\delta^{13}C$ measurements incorporating both accuracy and reproducibility is ± 0.5‰.[8,9]

Stable Chlorine Isotope Analysis.

Samples for $\delta^{37}Cl$ analysis were prepared in the same way as for $\delta^{13}C$ analysis. Chlorine isotope measurements were performed on a Neptune MC-ICPMS (Thermo Fisher Scientific, Germany) operated with conditions as described previously.[10,11] A Thermo Scientific Trace 1310 gas chromatographic (GC) system equipped with a flame ionization detector (FID) was interfaced with the MC-ICPMS to separate organics. Samples were injected to the GC system using a gastight syringe (Hamilton) into a split/splitless injector kept at 250°C with a split ratio of 1:10 or 1:5 and a helium carrier gas flow of 2 mL min^{-1}. A ZB-1 column (Phenomenex, 60 m x 0.32 mm x 1 µm film) was used that was held isothermally at 80°C. After separation, compounds entered the MC-ICPMS plasma via a Thermo Elemental Transferline AE2080 (Aquitaine Electronique, France) heated to 250°C to avoid condensation. Linear regression of the intensity recorded for mass 37 vs. mass 35 at each time point was used to determine the isotopic ratio, where the slope of the best fit line is equal to $^{37}Cl/^{35}Cl$.[12,13]

Raw $\delta^{37}Cl$ values of the samples were obtained by referencing all compounds versus a methyl chloride working standard which was injected before and after each sequence of samples. In a second step, raw $\delta^{37}Cl$ values were converted to the international reference scale for chlorine (SMOC) by applying a two-point calibration.[14–16] Three in-house standards characterized offline against SMOC (measured using DI-IRMS) were used for this procedure: methyl chloride (MC, $\delta^{37}Cl = +6.02‰$), and two different trichloroethenes TCE2 ($\delta^{37}Cl = -1.19‰$), and TCE6 ($\delta^{37}Cl = +2.17‰$).[14] Before the measurement of samples started, a sequence of 3–5 in-house standards were injected daily to determine the scale difference compared to DI-IRMS which is considered to present the SMOC scale (Eq. S1):

$$\delta^{37}Cl_{SMOC} = a * \delta^{37}Cl_{RAW} + b \qquad \text{Eq. S1}$$

The slope *a* (scale expansion factor) and intercept *b* (additive correction factor) represent the linear regression of the measured $\delta^{37}Cl_{RAW}$ of MC and TCE2 (both served as isotopic anchors) plotted versus the offline characterized $\delta^{37}Cl_{SMOC}$ ("true values") of these compounds. A third standard (TCE6) was used to evaluate the accuracy of this correction procedure, where the two-point calibrated $\delta^{37}Cl_{SMOC}$ of TCE6 had to agree with the offline value of +2.17‰ within 0.2‰. The maximum precision (1σ) observed for sample and control measurements was 0.28.

Calculations.

Eq. 3 in the main text was used to calculate ε_{bulk} values for biotransformation of 1,1,1-TCA and 1,1-DCA. Fraction remaining values at each time point were corrected for mass removal using the stepwise method of Buchner et al.[17] The maximum effect of repetitive sampling (calculated as the difference between corrected and uncorrected f values, maximum of 7% at final time point) was in all cases equal to the uncertainty of f (7%, calculated by propagating analytical uncertainty for composition analysis through the calculation of f). The f values calculated using the SW correction method were taken forward for calculation. Bulk ε_E values for each sample bottle were calculated by plotting $\ln[(\delta^{13}C_t+1)/(\delta^{13}C_0+1)]$ vs. ln f and determining the slope of the linear regression (m), where m = ε_{bulk} (in ‰).[18,19] Data from all replicate bottles in each experiment agreed within 95% confidence intervals, and thus all data for each experiment were combined to calculate ε_E.[20] AKIE values were calculated using Eq. 4 in the main text.

Dual-isotope plots were regressed using the York method[21] based on recommendations from Ojeda et al. [22,23] Goodness of fit of York-regressed slopes were evaluated using MSWD and p-values (see detailed discussion in Ojeda et al.[22,23]). Statistical tests (z-tests) were used to compare regression slopes.[22]

Sequence and Structure Comparison of CfrA between with Previously Published Results.

The updated CfrA sequence was obtained through BLAST[24], using the published *cfrA* sequence (Accession: AFV05253[25]) as a query against a custom database created from recent ACT-3/CF metagenomic data[26]. The query *cfrA* sequence was aligned to the top BLAST hit (Bit score: 2521.8, HSP score: 1365, E-value: 0, Mismatches: 2) using MUSCLE[27] to find mutations. Protein structure models of each sequence were created using SWISS-MODEL using the PCE reductive dehalogenase (PDB ID: 5m2g[28]) as a target sequence[29]. Structures were compared using PyMOL to visualize the mutation locations.[30]

5. Carbon isotope Results for 1,1,1-TCA Biotransformation by ACT-3 between studies

As described in the main text, significant differences are observed in the $AKIE_C$ reported here compared to results from Sherwood Lollar et al.[7] and Douglas[31] (Table S1). Sherwood Lollar et al.[7] ruled out diffusion through the cell membrane as a mass transfer step through experiments using whole cells and cell free extracts, however other mass transfer processes (e.g., transport due to radial or linear diffusion[32]) can theoretically impact the bioavailability of substrates and contribute to masking effects.[33–35] The $\varepsilon_{C\ bulk}$ values show a trend towards more negative values as the initial bulk substrate concentration (C_{bulk}, aqueous concentration) increases relative to the K_m (C_{bulk}/K_m; Figure 4). This is consistent with the effects of mass transfer limitations that affect the concentration of substrate available for biotransformation.[33,36] Based on Michaelis-Menten kinetics, when bioavailable concentrations decrease sufficiently relative to the K_M for an enzymatic reaction, the reaction kinetics change from zero-order kinetics (where increased substrate concentrations do not increase rate due to enzyme saturation with substrate) to first-order kinetics (where the rate of the enzyme-substrate binding step is decreased due to decreased

substrate concentrations).[33,36] The reaction order of the enzyme-substrate binding step is then dependent on the substrate concentration, and thus decreasing bioavailable substrate concentrations could act to decrease the rate of enzyme-substrate binding. This in turn can cause increased masking at lower substrate concentrations due to an interplay of mass transport and enzyme-substrate binding step as both become rate-limiting (increased rate-limitation in the overall enzymatic pathway relative to the transformation step) as concentrations decrease. Further degradation experiments with this culture are required with varying 1,1,1-TCA concentrations to test this hypothesis.

Table S1. Comparison of carbon isotope fractionation (ε_C and $AKIE_C$) in this work and from previous studies using ACT-3 TCA/EL with 1,1,1-TCA or ACT-3 DCA/EL with 1,1-DCA as substrate. Uncertainty of ε_C was calculated by 95% confidence interval (C.I.) on the slope of the Rayleigh model (**Error! Reference source not found.** of main text). Uncertainty of $AKIE_C$ values was calculated by propagating error through the AKIE calculation (Eq. 4 of main text).

	**This work**	**Douglas (2015)**[31]	**Sherwood Lollar et al. (2010)**[7]	
			**Whole Cell (WC)**	**Cell-free extract (CFE)**
**ACT-3 1,1,1-TCA/EL**				
ε_C ± 95% C.I. (‰)	-5.8 ± 0.8	-3.0 ± 0.2	-1.8 ± 0.3	-0.8 ± 0.3
$AKIE_C$	1.0118 ± 0.0017	1.00603 ± 0.00041	1.00361 ± 0.00061	1.00160 ± 0.00061
**ACT-3 1,1-DCA/EL**				
ε_C ± 95% C.I. (‰)	-8.9 ± 1.0	NA	-10.5 ± 0.6	-7.9 ± 0.9
$AKIE_C$	1.0181 ± 0.0022	NA	1.0215 ± 0.0013	1.0161 ± 0.0019

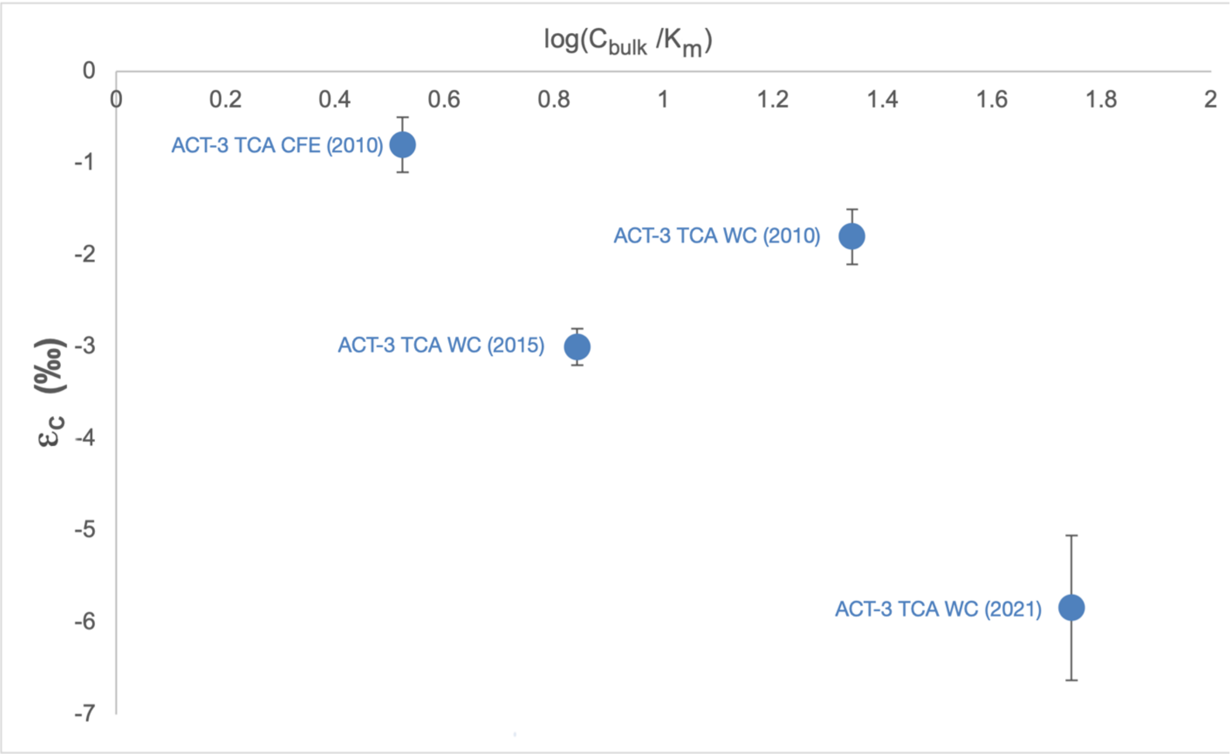

Figure S4. ε_C vs. $\log(C_{bulk}/K_m)$ for 1,1,1-TCA degradation. K_m values are from Grostern et al.[37] for ACT-3 TCA with 1,1,1-TCA in cell suspensions (18 ± 3 μM) and cell-free extracts (45 ± 18 μM) and for ACT-3 DCA with 1,1-DCA in cell suspensions (127 ± 27 μM) and cell-free extracts (413 ± 64 μM). Error bars show 95% C.I. for ε_C. ε_C values are presented in Figure 2 of main text. Data is shown for whole cells (WC) and cell-free extracts (CFE). The year indicates the year of the study where data was originally published, including C_{bulk} values, where 2015 indicates Douglas[31], 2010 indicates Sherwood Lollar et al.[7], and 2021 indicates this work.

The second hypothesis could be related to an amino acid mutation in CfrA. Recent sequencing data (sampled in Nov 2020) of CfrA shows mutations in two locations (at L154R and V244A) on the enzyme compared to data from its first sequence published in 2012 (sampled in May, 2010; Accession: AFV05253[25]).[38] Based on homology modelling, where the amino acid

sequence is compared to another sequence with a known structure in order to develop a model protein structure, L154R is in a location far away from the binding site, while V244A is close to the binding site (shown in Figure S5). Amino acid mutations in or proximal to the binding site of the enzyme can affect how enzyme binding to substrate due to changes in their interactions (e.g., through increased or decreased hydrogen bonding). As few as one or two mutations in the active site can have a major impact on dehalogenase activity[39]. Changes in these interactions could affect the kinetics of binding to and dissociation of the enzyme-substrate complex, and thus changes in the extent of masking could arise from differences in k_{-1}. These interactions could also stabilize the transition state of the transformation step, acting on k_2. Thus, the second reasonable mechanism for changes in isotope fractionation between this work and previous work (Table S2) is that differences in the interactions between the enzyme and substrate (e.g., through transition state stabilization, enzyme-substrate binding) resulted from the V244A mutation. Further experiments are required using mutant enzymes to investigate the effects of amino acid substitutions at these residues on carbon and chlorine isotope fractionation.

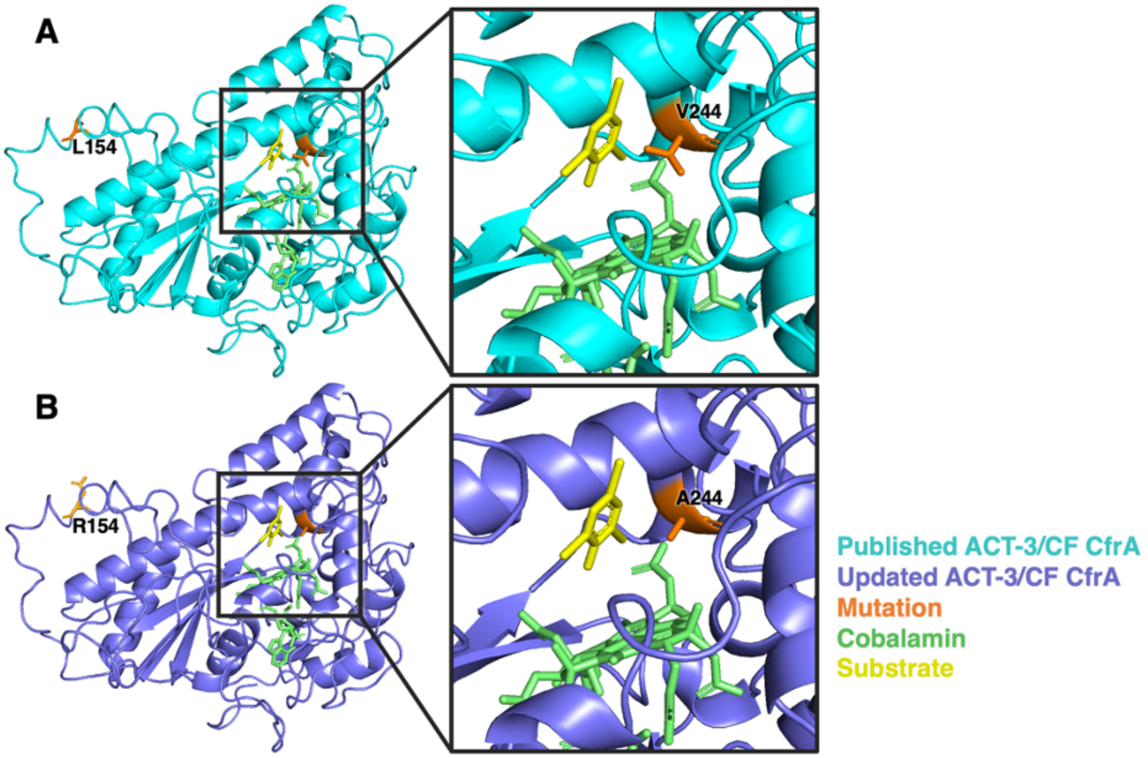

Figure S5. Homology model showing amino acid mutation in the RDase CfrA at location V244A. Homology model from when metagenome was originally sequenced is shown in (A), with valine at this site (V244). An updated model based on sequencing data from 2020[26] is shown in (B) with alanine (A244) at this site. The location of the amino acid mutation is shown in orange. The cobamide cofactor (green) and substrate used for crystallization in the template structure (yellow) are overlain with the CfrA model to demonstrate he enzyme's active site.

6. AKIE Values for Biotransformation of 1,1,1-TCA and 1,1-DCA by ACT-3

Table S2. $AKIE_C$ and $AKIE_{Cl}$ values calculated using Eq. 4. Error was calculated by propagating error through the AKIE calculations (Eq. 4). Theoretical maximum isotope effects can be estimated using semiclassical Streitwieser limits[40] for carbon (1.03) and chlorine (1.007).

	$AKIE_C$	$AKIE_{Cl}$
ACT-3/1,1,1-TCA	1.0118 ± 0.0017	1.00838 ± 0.00079
ACT-3/1,1-DCA	1.0181 ± 0.0022	1.00872 ± 0.00081

$AKIE_{Cl}$ and $AKIE_C$ values for 1,1,1-TCA are approximately equal in magnitude (Table S2). Given the greater relative mass difference between ^{13}C and ^{12}C compared to ^{37}Cl and ^{35}Cl, primary isotope effects are generally expected to be larger for carbon than for chlorine for a given reaction mechanism. While the $AKIE_{Cl}$ for 1,1,1-TCA (1.0084 ± 0.0008) approaches the semiclassical Streitwieser limits for chlorine (KIE_{Cl} = 1.013) assuming 50% bond cleavage in the transition state[25], the calculated $AKIE_C$ (1.0118 ± 0.0017) is considerably lower than the theoretical KIE_C of 1.03 (see earlier discussion). This similarity in magnitude of $AKIE_{Cl}$ and $AKIE_C$ may indicate significant secondary chlorine isotope effects, as discussed above and previously proposed in studies of abiotic 1,1,1-TCA transformation.[27] Because AKIE calculations typically assume that observed bulk isotope fractionation arises solely from primary effects, the presence of significant secondary isotope effects[44] can result in overestimated AKIE values.

7. Proposed Pathway for Hydrogenolysis of 1,1,1-TCA and 1,1-DCA by CfrA and DcrA, respectively

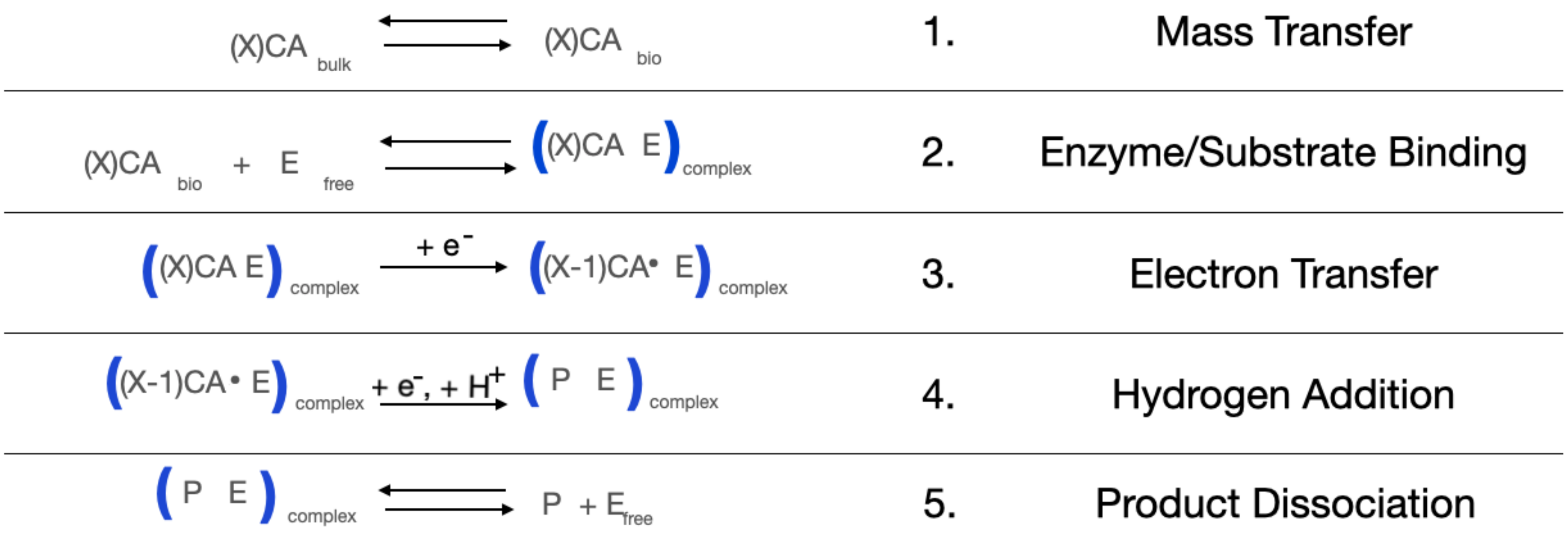

Figure S6. Overall pathway proposed for hydrogenolysis of the chlorinated alkanes 1,1,1-TCA and 1,1-DCA (denoted as CA) by enzymes (E) CfrA and DcrA, respectively. The number of chorines is represented by 'X', and the dechlorinated product is indicated by '(X-1)'. A single electron is shown as '●' Double arrows indicate hypothesized equilibrium steps, while single arrows indicate unidirectional steps.

8. Parent and Product $\delta^{37}Cl$ trends for chloroform (CF) biotransformation to dichloromethane (DCM)

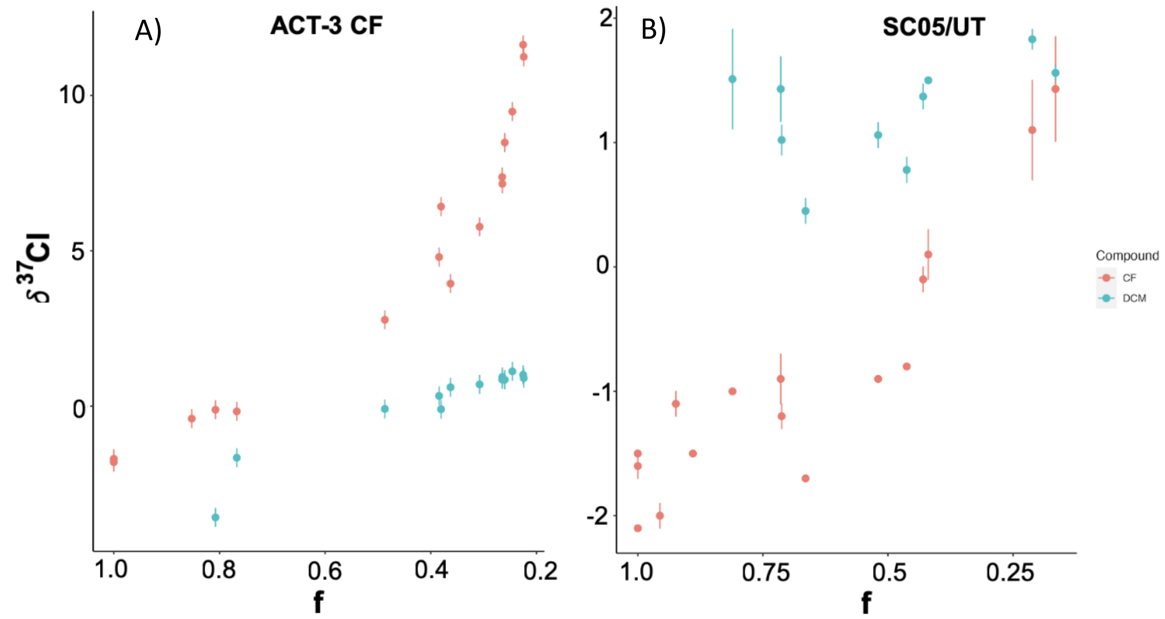

Figure S7. $\delta^{37}Cl$ CF transformation to DCM by A) ACT-3, where parent and product isotope trends consistent with the Rayleigh model are observed and B) SC05-UT (also known as KB-1® Plus CF), where product (DCM) $\delta^{37}Cl$ are enriched in ^{37}Cl relative to the substrate (CF). Data is from experiments published in Phillips et al.[41] Error bars on the dual plots represent analytical uncertainty for each isotope system, determined by sample and standard reproducibility.

Table S3. Estimated primary and secondary ε_{Cl} in 1,1,1-TCA and 1,1-DCA calculated following the approach described by Cretnik et al. (2014).

	**1,1,1-TCA Experiment**	**1,1-DCA Experiment**
$\varepsilon_{primary}$ (‰)	-20.7 ± 15.1	-7.0 ± 7.9
$\varepsilon_{secondary}$ (‰)	+4.1 ± 1.8	+3.3 ± 6.1

References

(1) Elsner, M.; Cwiertny, D. M.; Roberts, A. L.; Sherwood Lollar, B. 1,1,2,2-Tetrachloroethane Reactions with OH-, Cr(II), Granular Iron, and a Copper–iron Bimetal: Insights from Product Formation and Associated Carbon Isotope Fractionation. *Environ. Sci. Technol.* 2007, *41* (11), 4111–4117. https://doi.org/10.1021/es063040x.

(2) Palau, J.; Shouakar-Stash, O.; Hunkeler, D. Carbon and Chlorine Isotope Analysis to Identify Abiotic Degradation Pathways of 1,1,1-Trichloroethane. *Environ. Sci. Technol.* 2014, *48* (24), 14400–14408. https://doi.org/10.1021/es504252z.

(3) Broholm, M. M.; Hunkeler, D.; Tuxen, N.; Jeannottat, S.; Scheutz, C. Stable Carbon Isotope Analysis to Distinguish Biotic and Abiotic Degradation of 1,1,1-Trichloroethane in Groundwater Sediments. *Chemosphere* 2014, *108*, 265–273. https://doi.org/10.1016/j.chemosphere.2014.01.051.

(4) Fennelly, J.; Roberts, A. L. Reaction of 1,1,1-Trichloroethane with Zero-Valent Metals and Bimetallic Reductants. *Environ. Sci. Technol.* 1998, *32* (13), 1980–1988.

(5) Song, H.; Carraway, E. R. Reduction of Chlorinated Methanes by Nano-Sized Zero-Valent Iron. Kinetics, Pathways, and Effect of Reaction Conditions. *Environ. Eng. Sci.* 2006, *23* (2), 272–284. https://doi.org/10.1089/ees.2006.23.272.

(6) Cretnik, S.; Bernstein, A.; Shouakar-Stash, O.; Löffler, F.; Elsner, M. Chlorine Isotope Effects from Isotope Ratio Mass Spectrometry Suggest Intramolecular C-Cl Bond Competition in Trichloroethene (TCE) Reductive Dehalogenation. *Molecules* 2014, *19* (5), 6450–6473. https://doi.org/10.3390/molecules19056450.

(7) Sherwood Lollar, B.; Hirschorn, S.; Mundle, S. O. C.; Grostern, A.; Edwards, E. A.; Lacrampe-Couloume, G. Insights into Enzyme Kinetics of Chloroethane Biodegradation Using Compound Specific Stable Isotopes. *Environ. Sci. Technol.* 2010, *44* (19), 7498–7503. https://doi.org/10.1021/es101330r.

(8) Sherwood Lollar, B.; Hirschorn, S. K.; Chartrand, M. M. G.; Lacrampe-Couloume, G. An Approach for Assessing Total Instrumental Uncertainty in Compound-Specific Carbon Isotope Analysis: Implications for Environmental Remediation Studies. *Anal. Chem.* 2007, *79* (9), 3469–3475. https://doi.org/10.1021/ac062299v.

(9) Horst, A.; Lacrampe-Couloume, G.; Sherwood Lollar, B. Compound-Specific Stable Carbon Isotope Analysis of Chlorofluorocarbons in Groundwater. *Anal. Chem.* 2015, *87* (20), 10498–10504. https://doi.org/10.1021/acs.analchem.5b02701.

(10) Horst, A.; Renpenning, J.; Richnow, H.; Gehre, M. Compound Specific Stable Chlorine Isotopic Analysis of Volatile Aliphatic Compounds Using Gas Chromatography Hyphenated with Multiple Collector Inductively Coupled Plasma Mass Spectrometry. *Anal. Chem.* 2017, *89*, 9131–9138. https://doi.org/10.1021/acs.analchem.7b01875.

(11) Renpenning, J.; Horst, A.; Schmidt, M.; Gehre, M. Online Isotope Analysis of 37Cl/35Cl Universally Applied for Semi-Volatile Organic Compounds Using GC-MC-ICPMS. *J. Anal. At. Spectrom.* 2018, *33* (2), 314–321. https://doi.org/10.1039/c7ja00404d.

(12) Fietzke, J.; Frische, M.; Hansteen, T. H.; Eisenhauer, A. A Simplified Procedure for the Determination of Stable Chlorine Isotope Ratios (δ37Cl) Using LA-MC-ICP-MS. *J. Anal. At. Spectrom.* 2008, *23* (5), 769–772. https://doi.org/10.1039/b718597a.

(13) Epov, V. N.; Rodriguez-Gonzalez, P.; Sonke, J. E.; Tessier, E.; Amouroux, D.; Bourgoin, L. M.; Donard, O. F. X. Simultaneous Determination of Species-Specific Isotopic

Composition of Hg by Gas Chromatography Coupled to Multicollector ICPMS. *Anal. Chem.* 2008, *80* (10), 3530–3538. https://doi.org/10.1021/ac800384b.

(14) Renpenning, J.; L. Hitzfeld, K.; Gilevska, T.; Nijenhuis, I.; Gehre, M.; Richnow, H.-H. Development and Validation of an Universal Interface for Compound-Specific Stable Isotope Analysis of Chlorine (37Cl/35Cl)) by GC-High-Temperature Conversion (HTC)-MS/IRMS. *Anal. Chem.* 2015, *87* (5), 2832–2839. https://doi.org/10.1021/ac504232u.

(15) Coplen, T. B. Normalization of Oxygen and Hydrogen Isotope Data. *Chemical Geology: Isotope Geoscience Section* 1988, *72* (4), 293–297. https://doi.org/10.1016/0168-9622(88)90042-5.

(16) Paul, D.; Skzypek, G.; Forizas, I. Normalization of Measured Stable Isotopic Compositions to Isotope Reference Scales - a Review. *Rapid Communications in Mass Spectrometry* 2007, *21*, 3006–3014. https://doi.org/10.1002/rcm.

(17) Buchner, D.; Jin, B.; Ebert, K.; Rolle, M.; Elsner, M.; Haderlein, S. B. Experimental Determination of Isotope Enrichment Factors - Bias from Mass Removal by Repetitive Sampling. *Environ. Sci. Technol.* 2017, *51* (3), 1527–1536. https://doi.org/10.1021/acs.est.6b03689.

(18) Scott, K. M.; Lu, X.; Cavanaugh, C. M.; Liu, J. S. Optimal Methods for Estimating Kinetic Isotope Effects from Different Forms of the Rayleigh Distillation Equation. *Geochim. Cosmochim. Acta* 2004, *68* (3), 433–442. https://doi.org/10.1016/S0016-7037(03)00459-9.

(19) Hunkeler, D.; Meckenstock, R. U.; Sherwood Lollar, B.; Schmidt, T. C.; Wilson, J. T.; Schmidt, T.; Wilson, J. A Guide for Assessing Biodegradation and Source Identification of Organic Ground Water Contaminants Using Compound Specific Isotope Analysis (CSIA). *Oklahoma, USA, US EPA* 2008.

(20) Mundle, S. O. C.; Vandersteen, A. A.; Lacrampe-Couloume, G.; Kluger, R.; Sherwood Lollar, B. Pressure-Monitored Headspace Analysis Combined with Compound-Specific Isotope Analysis to Measure Isotope Fractionation in Gas-Producing Reactions. *Rapid Communications in Mass Spectrometry* 2013, *27* (15), 1778–1784. https://doi.org/10.1002/rcm.6625.

(21) York, D.; Evensen, N. M.; Martínez, M. L.; de Basabe Delgado, J. Unified Equations for the Slope, Intercept, and Standard Errors of the Best Straight Line. *Am. J. Phys.* 2004, *72* (3), 367–375. https://doi.org/10.1119/1.1632486.

(22) Ojeda, A.; Phillips, E.; Mancini, S.; Sherwood Lollar, B. Sources of Uncertainty in Biotransformation Mechanistic Interpretations and Remediation Studies Using CSIA. *Anal. Chem. 91* (14), 9147–9153. https://doi.org/10.1021/acs.analchem.9b01756.

(23) Ojeda, A.S., Zheng, J., Phillips, E., Sherwood Lollar, B. Quantifying Regression Bias for Multi-Element Isotope Analysis in Contaminant Hydrogeology. *Talanta* 2021, *226*.

(24) Altschul, S. F.; Gish, W.; Miller, W.; Myers, E. W.; Lipman, D. J. Basic Local Alignment Search Tool. *J. Mol. Biol.* 1990, *215* (3), 403–410. https://doi.org/10.1016/S0022-2836(05)80360-2.

(25) Tang, S.; Gong, Y.; Edwards, E. A. Semi-Automatic In Silico Gap Closure Enabled De Novo Assembly of Two *Dehalobacter* Genomes from Metagenomic Data. *PLoS One* 2012, *7* (12). https://doi.org/10.1371/journal.pone.0052038.

(26) Bulka, O.; Edwards, E. Metagenome from ACT-3/CF: An Anaerobic Chloroform-Degrading Microbial Community. *Microbiol. Resour. Announc.* 2024, *13* (10). https://doi.org/10.1128/mra.00674-24.

(27) Edgar, R. C. MUSCLE: Multiple Sequence Alignment with High Accuracy and High Throughput. *Nucleic Acids Res.* 2004, *32* (5), 1792–1797. https://doi.org/10.1093/nar/gkh340.
(28) Kunze, C.; Bommer, M.; Hagen, W. R.; Uksa, M.; Dobbek, H.; Schubert, T.; Diekert, G. Cobamide-Mediated Enzymatic Reductive Dehalogenation via Long-Range Electron Transfer. *Nat. Commun.* 2017, *8*. https://doi.org/10.1038/ncomms15858.
(29) Waterhouse, A.; Bertoni, M.; Bienert, S.; Studer, G.; Tauriello, G.; Gumienny, R.; Heer, F. T.; De Beer, T. A. P.; Rempfer, C.; Bordoli, L.; Lepore, R.; Schwede, T. SWISS-MODEL: Homology Modelling of Protein Structures and Complexes. *Nucleic Acids Res.* 2018, *46* (W1), W296–W303. https://doi.org/10.1093/nar/gky427.
(30) Schrödinger LLC. The PyMOL Molecular Graphics System.
(31) Douglas, L. M. Investigating Controls on Variation in Isotopic Fractionation during Biodegradation of Chlorinated Ethenes and Ethanes, 2015. https://doi.org/10.1088/1751-8113/44/8/085201.
(32) Bosma, T. N. P.; Middeldorp, P. J. M.; Schraa, G.; Zehnder, A. J. B. Mass Transfer Limitation of Biotransformation: Quantifying Bioavailability. *Environ. Sci. Technol.* 1997, *31* (1), 248–252. https://doi.org/10.1021/es960383u.
(33) Kampara, M.; Thullner, M.; Richnow, H. H.; Harms, H. Impact of Bioavailability Restrictions on Microbially Induced Stable. *Environ. Sci. Technol.* 2008, *42* (17), 6552–6558.
(34) Thullner, M.; Kampara, M.; Richnow, H. H.; Harms, H.; Wick, L. Y. Impact of Bioavailability Restrictions on Microbially Induced Stable Isotope Fractionation. 1. Theoretical Calculation. *Environ. Sci. Technol.* 2008, *42* (17), 6544–6551. https://doi.org/10.1021/es702782c.
(35) Ehrl, B. N.; Kundu, K.; Gharasoo, M.; Marozava, S.; Elsner, M. Rate-Limiting Mass Transfer in Micropollutant Degradation Revealed by Isotope Fractionation in Chemostat. *Environ. Sci. Technol.* 2019, *53* (3), 1197–1205. https://doi.org/10.1021/acs.est.8b05175.
(36) Thullner, M.; Centler, F.; Richnow, H. H.; Fischer, A. Quantification of Organic Pollutant Degradation in Contaminated Aquifers Using Compound Specific Stable Isotope Analysis - Review of Recent Developments. *Org. Geochem.* 2012, *42* (12), 1440–1460. https://doi.org/10.1016/j.orggeochem.2011.10.011.
(37) Grostern, A.; Chan, W. W. M.; Edwards, E. A. 1,1,1-Trichloroethane and 1,1-Dichloroethane Reductive Dechlorination Kinetics and Co-Contaminant Effects in a *Dehalobacter*-Containing Mixed Culture. *Environ. Sci. Technol.* 2009, *43* (17), 6799–6807. https://doi.org/10.1021/es901038x.
(38) Tang, S.; Wang, P. H.; Higgins, S. A.; Löffler, F. E.; Edwards, E. A. Sister *Dehalobacter* Genomes Reveal Specialization in Organohalide Respiration and Recent Strain Differentiation Likely Driven by Chlorinated Substrates. *Front. Microbiol.* 2016, *7* (FEB), 1–14. https://doi.org/10.3389/fmicb.2016.00100.
(39) Picott, K. J.; Bowers, C. M.; Edwards, E. A. Deciphering Reductive Dehalogenase Specificity through Targeted Mutagenesis of Chloroalkane Reductases. *Appl. Environ. Microbiol.* 2025, *91* (3). https://doi.org/10.1128/aem.01501-24.
(40) Huskey, W. P. *Enzyme Mechanism from Isotope Effects*; Cook, P. F., Ed.; CRC Press: Boca Raton, 1991.
(41) Phillips, E.; Bulka, O.; Picott, K.; Kümmel, S.; Edwards, E. A.; Nijenhuis, I.; Gehre, M.; Dworatzek, S.; Webb, J.; Sherwood Lollar, B. Investigation of Active Site Amino Acid

Influence on Carbon and Chlorine Isotope Fractionation during Reductive Dechlorination. *FEMS Microbiol. Ecol.* 2022, *98* (8). https://doi.org/10.1093/femsec/fiac072.
